# Phase-based fast 3D high-resolution quantitative T_2_ MRI in 7 T human brain imaging

**DOI:** 10.1038/s41598-022-17607-z

**Published:** 2022-08-18

**Authors:** Amir Seginer, Rita Schmidt

**Affiliations:** 1 Siemens Healthcare Ltd, Rosh Ha’ayin, Israel; 2 Department of Brain Sciences, Weizmann Institute of Science, Rehovot, Israel; 3 The Azrieli National Institute for Human Brain Imaging and Research, Weizmann Institute of Science, Rehovot, Israel

**Keywords:** Biomedical engineering, Imaging techniques

## Abstract

Magnetic resonance imaging (MRI) is a powerful and versatile technique that offers a range of physiological, diagnostic, structural, and functional measurements. One of the most widely used basic contrasts in MRI diagnostics is transverse relaxation time (T_2_)-weighted imaging, but it provides only *qualitative* information. Realizing *quantitative* high-resolution T_2_ mapping is imperative for the development of personalized medicine, as it can enable the characterization of diseases progression. While ultra-high-field (≥ 7 T) MRI offers the means to gain new insights by increasing the spatial resolution, implementing fast quantitative T_2_ mapping cannot be achieved without overcoming the increased power deposition and radio frequency (RF) field inhomogeneity at ultra-high-fields. A recent study has demonstrated a new phase-based T_2_ mapping approach based on fast steady-state acquisitions. We extend this new approach to ultra-high field MRI, achieving quantitative high-resolution 3D T_2_ mapping at 7 T while addressing RF field inhomogeneity and utilizing low flip angle pulses; overcoming two main ultra-high field challenges. The method is based on controlling the coherent transverse magnetization in a steady-state gradient echo acquisition; achieved by utilizing low flip angles, a specific phase increment for the RF pulses, and short repetition times. This approach simultaneously extracts both T_2_ and RF field maps from the phase of the signal. Prior to in vivo experiments, the method was assessed using a 3D head-shaped phantom that was designed to model the RF field distribution in the brain. Our approach delivers *fast* 3D whole brain images with submillimeter resolution without requiring special hardware, such as multi-channel transmit coil, thus promoting high usability of the ultra-high field MRI in clinical practice.

## Introduction

Non-invasive biomedical imaging provides high-impact medical diagnostics and offers an ideal means of promoting preventative medicine. This is indeed the case when it comes to ultra-high field (≥ 7 T) Magnetic Resonance Imaging (MRI)^1–3^. One high-value diagnostic MRI method is based on estimating the T_2_ relaxation time of tissues—either T_2_-weighted^4^ imaging or quantitative-T_2_ mapping^5–7^. T_2_-weighted MRI of the brain is one of the most widely employed routine diagnostic methods in cancer and neurodegenerative diseases. It is essential for the detection of hyperintense lesions pronounced in demyelinating diseases, such as multiple sclerosis^8–10^, and in the monitoring of disease progression^9^. In multiple sclerosis, improved precision at early stages of lesion formation would allow their clear categorization and aid in developing new tools to delay or eliminate the relapse. Recent studies at 7 T MRI have shown that we can detect smaller lesions than previously possible and so better monitor disease progression^8^. However, the robust characterization of disease progression with MRI requires quantitative T_2_ mapping, the use of which in clinics is impeded by its long scan duration. Novel fast methods^11–13^ encounter extra challenges in ultra-high field MRI among which are the severe RF field inhomogeneity^14^, which reduces the accuracy of the quantification, and the increased power deposition that results in prolonged scan duration. Common T_2_ methods are especially prone to the above drawbacks since they are spin-echo-based, requiring refocusing pulses that are high in Specific Absorption Rate (SAR)^15^ and whose effectiveness is sensitive to RF field inhomogeneity.

Recent studies have proposed another solution—called Magnetic Resonance Fingerprinting (MRF)^16,17^. This method allows parametric MR mapping (including T_1_ and T_2_ maps), thus eliminating the dependence on the specific scan parameter or scanner. However, MRF is not easily translated into ultra-high field MRI, since overcoming the RF field inhomogeneity further complicates the acquired dataset^17^. Designs based on the steady-state gradient echo (GRE) pulse sequences offer a plethora of pathways toward multi-contrast fast acquisitions, among which are simultaneous multi-parametric acquisitions^18,19^ as well as a design for T_1_ and T_2_ weighted images in highly inhomogeneous static magnetic fields^20^. These include DESPOT2^21^ and phase-cycled balanced steady-state free precession^22–24^ (bSSFP). A method called TESS^19,25^ shows promising results for T_2_ mapping without RF field dependence, however, currently it was demonstrated only as a 2D implementation for brain imaging^26^. Finally, a method analyzing the complex signal of a set of unbalanced GRE scans at 7 T gave T_1_ and T_2_ maps^18^, but included a long total scan duration (16:36 min) and used parallel transmission to mitigate the transmit field inhomogeneity.

**Figure 1:**
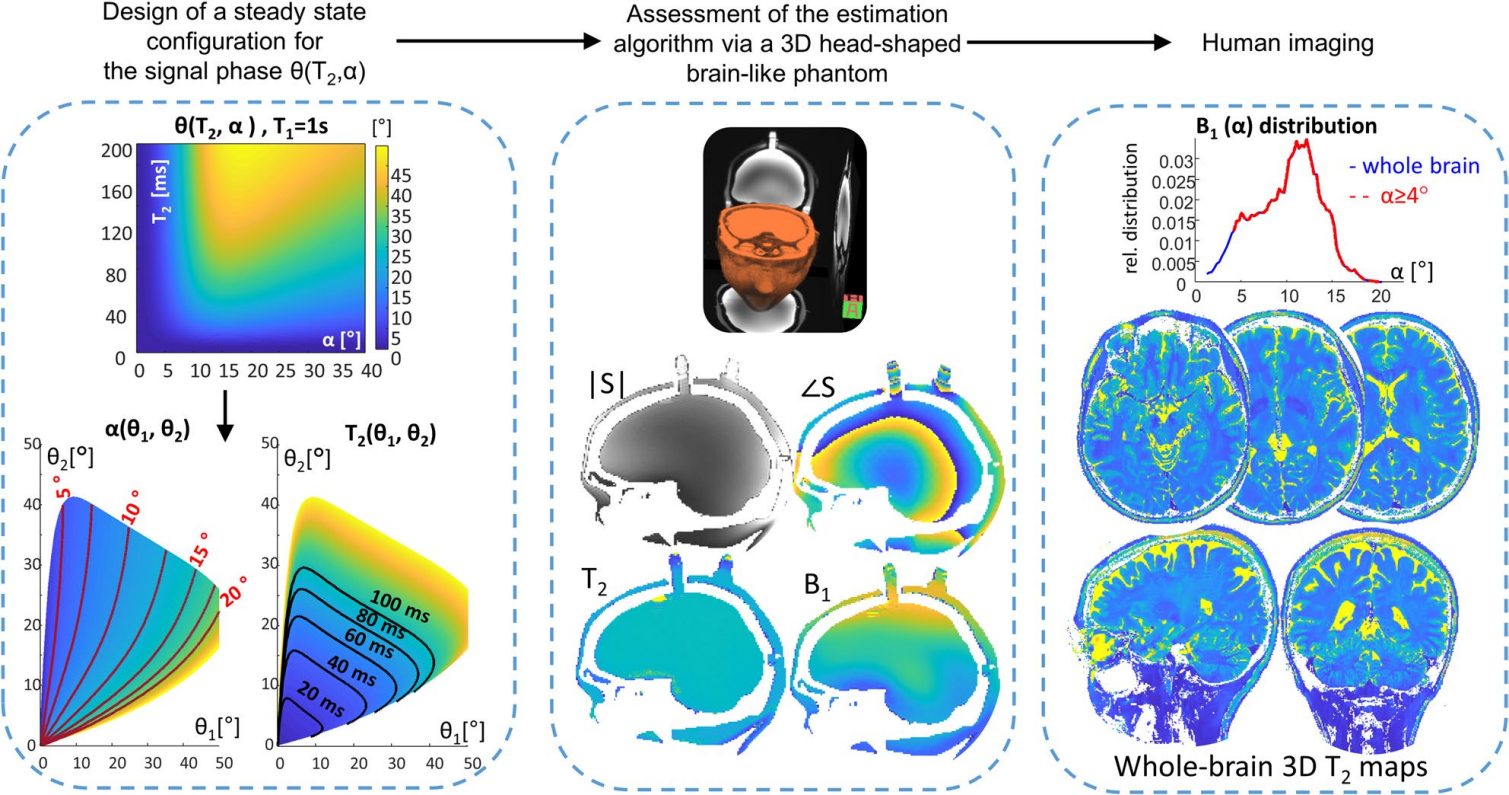
Schematics of the steady state method for T_2_ and RF field estimation—its design and verification. Starting from simulations, through assessment of the estimation algorithm, via a 3D head-shaped brain-like phantom, to human imaging. Left—a design of a steady-state configuration based on Bloch simulations that provides θ(T_2_,α) for specific φ_inc,_ which was thereafter utilized to generate T_2_ and α in the 2D space (θ_1_, θ_2_). The new space allows to extract T_2_ and α from θ_1_ and θ_2_. Center—the estimation algorithm was assessed via simulations and brain-like phantom measurements. In these measurements, a realistic signal $S$ was acquired, providing |S| and $\angle S$, from which the T_2_ and α (or B_1_ distribution) were estimated. Right—human imaging at 7 T MRI provided high-resolution whole-brain T_2_ maps, while coping with the B_1_ distribution.

Recently, a new method was introduced based on a steady-state spoiled gradient-echo (SGRE) acquisition that utilizes low flip angles and short repetition times (TRs) to obtain T_2_ maps at 3 T MRI^27^, assuming a uniform and a priori known flip angle. While most of the GRE-based studies have focused on magnitude images^20,21,28^, in this study, phase information was highlighted, which offers a new and attractive method for T_2_ mapping. Building on this work we elucidate the dependence of the phase-based method on the (unknown) excitation flip angle in addition to the RF pulse phase, with an eye to design an approach suited for T_2_ mapping of the brain at 7 T. This new extension to the steady state method includes both T_2_ and RF field estimation and is designed to cover the relevant flip angle range arising in the brain due to the RF field inhomogeneity at 7 T MRI (see Fig. 1). The advantage of this approach is its ability to simplify the signal dependencies and reduce the confounding variables. This includes the removal of the static magnetic field (B_0_) dependence and a reduced dependence on the longitudinal relaxation time (T_1_).

Building on the phase-based approach by Wang^27,29^, which departs from the traditional concepts based on spin-echo, our extension introduces a new T_2_ mapping solution for ultra-high field MRI. This method can deliver quantitative T_2_ mapping at 7 T MRI without requiring any additional hardware—such as dielectric pads or multi-channel transmit coil—to reduce the RF field inhomogeneity. Another advantage of this method is that it enables whole-brain imaging with high acceleration factors, as it relies on a 3D k-space acquisition.

Our study comprised three main steps (Fig. 1). First, we conducted Bloch simulations of the generated steady state signal for different scan parameters (such as RF flip angle, RF phase, and TR). Then, we looked for parameter combinations that support the range of flip angles, due to the RF field inhomogeneity, in 7 T brain imaging. Next, we developed and assessed an efficient estimation algorithm. Lastly, we performed human brain imaging on a 7 T MRI scanner. The estimation algorithm was assessed on synthetic signals from the Bloch simulations, as well as on actual measurements, in a realistic setting, via a 3D head-shaped phantom, which was designed to model the RF field distribution in the brain^30^. For both the head-shaped phantom and the human brain imaging the phase-based method was compared with the gold-standard single-echo spin echo (SE–SE). Furthermore, we used the phase-based method to acquire whole-brain T_2_ maps with sub-millimeter resolution.

### Principles of the modified-SGRE sequence for simultaneous T_2_ and RF field mapping

**Figure 2:**
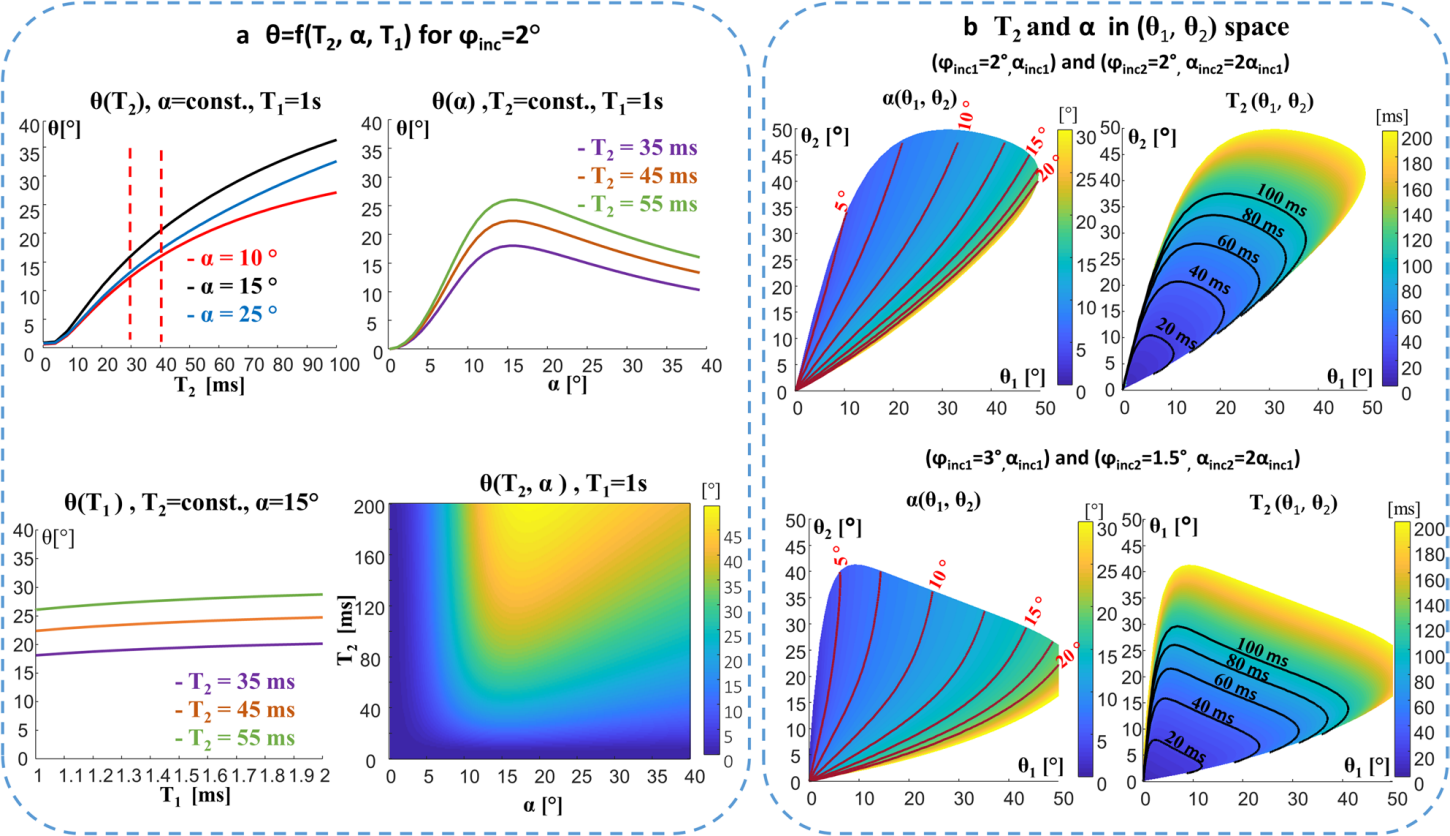
The phase of the steady-state signal as a function of T_2_ and the flip angle. (**a**) θ(T_2_, α, T_1_) dependence (Bloch simulation results) for a representative small φ_inc_ (φ_inc_ = 2°). The dependence on T_2_, α, and T_1_ is shown in 1D plots and in 2D. (**b**) T_2_ and α distributions in the new (θ_1_, θ_2_) 2D space. Two examples are shown: Top—(φ_inc1_ = 2°_,_ α_scan1_) with (φ_inc2_ = 2°_,_ α_scan2_ = 2α_scan1_). Bottom—(φ_inc1_ = 3°_,_ α_scan1_) with (φ_inc2_ = 1.5°_,_ α_scan2_ = 2α_scan1_). In each case, α (θ_1_, θ_2_) and T_2_ (θ_1_, θ_2_) are shown with the equi-T_2_ and equi-α lines.

The foundation for using phase increments during the RF pulse train was provided by Zur et al. in 1988^31,32^. They showed that an RF pulse train with a quadratic phase φ_RF_(n) = φ_inc_∙(n^2^ + n)/2 for the n-th pulse—using an appropriate φ_inc_ value in conjunction with a spoiling gradient—can achieve incoherent transverse magnetization, an effective spoiling better than simple gradient spoiling (φ_inc_ = 0 case). This is commonly called RF spoiling. Recent work by Wang^27^ at 3 T provided another keystone, in which the authors showed that small φ_inc_ values have the opposite effect; they introduce coherent transverse magnetization, where the phase of the signal possess a strong dependence on T_2_ (Fig. 2a). Figure 2a also shows the dependence of the phase of the signal on the excitation flip angle α. The α dependence curves have an extremum in the vicinity of 15°, i.e., the actual flip angle in that vicinity has a small effect on the phase. As the flip angle in the 3 T implementation was assumed to be given by the scan (due to relatively homogeneous RF field distribution), T_2_ values could be extracted solely from the phase of the signal.

In our study, however, the combined (T_2_, α) dependence of the signal’s phase θ was exploited to cope with the RF field inhomogeneity at ultra-high field MRI. Neglecting, for now, the small T_1_ dependence of the phase—for T_1_ values relevant to brain tissues at 7 T, see Fig. 2a—the phase θ of the signal depends on the T_2_ at the voxel and on the actual flip angle α there. This α is the target flip angle of the scan α_scan_ scaled by the RF field ratio at each voxel: α = α_scan_∙RF_ratio_, where RF_ratio_ is the normalized RF field distribution. As the phase θ(T_2_, α) (see Fig. 2a) is not a one-to-one map of (T_2_, α) to θ, at least two measurements, θ_1_ and θ_2_, are needed; thus defining a 2D space (θ_1_, θ_2_). To extract T_2_ and α from θ(T_2_,α), we need a convenient 2D space to represent T_2_ and α in each voxel_._ Based on the Bloch simulations, such a 2D space can be generated by two scans with two flip angles, α_scan1_ and α_scan2_ = R_FA_∙α_scan1_ (R_FA_ is a user set multiplication factor; for example, R_FA_ = 2). Furthermore, we found that varying φ_inc_ between the two scans—one scan with (φ_inc1,_ α_scan1_) and a second with (φ_inc2,_ α_scan2_ = R_FA_∙ α_scan1_)—provides greater flexibility in controlling the 2D (θ_1_, θ_2_) space and its mapping to (T_2_, α). Figure 2b shows that different combinations of phase increment and flip angle pairs can be useful to adjust the range of viable flip angles and the T_2_ of interest.

The two phase measurements, θ_1_ for scan parameters (φ_inc1_, α_scan1_) and θ_2_ for scan parameters (φ_inc2,_ α_scan2_), are functions of φ_inc1_, φ_inc2_, T_2_ and the (actual) flip angles, i.e., θ_1_ = θ(φ_inc1_, α_1_, T_2_) and θ_2_ = θ(φ_inc2_, α_2_, T_2_), where α_1_ and α_2_ are the actual flip angles. Although α_1_ and α_2_ are unknown, their ratio must obey α_2_/α_1_ = α_scan2_/α_scan1_ $\equiv$ R_FA_. Thus, renaming α_1_ as α, we have θ_1_ = θ(φ_inc1_, α, T_2_) and θ_2_ = θ(φ_inc2_, R_FA_∙α, T_2_), or in a shorthand notation θ_1_ = θ_1_(α, T2) and θ_2_ = θ_2_(α, T_2_), where the functions θ_1_() and θ_2_() contain the known φ_inc1_, φ_inc2_, and R_FA_ parameters. One can now map T_2_ and α to the new (θ_1_, θ_2_) 2D space, written as T_2_(θ_1_, θ_2_) and α(θ_1_, θ_2_). Figure 2b shows that equi-T_2_ and equi-α lines are nearly orthogonal, when we are well inside the “balloon” (the support region), which is an indication of the robust estimation for a given set of (θ_1_,θ_2_) there. At the “balloon” edges of low or high α values the solution is ill-posed and can provide more than one solution, thus increasing the variability and bias of the estimation at that region. Having now the simulated (θ_1_, θ_2_) 2D space for T_2_ and for α, one can point with any measured (θ_1meas._,θ_2meas._) to that space and provide the expected T_2_ and α values by a simple interpolation. This representation is useful to explore and characterize optimal choices of flip angles and φ_inc_ to achieve minimal variability and bias. Figure 2b shows that the set (φ_inc1_ = 3°_,_ α_scan1_) and (φ_inc2_ = 1.5°_,_ α_scan2_ = 2α_scan1_) covers a larger flip-angle range than set (φ_inc1_ = 2°_,_ α_scan1_) and (φ_inc2_ = 2°_,_ α_scan2_ = 2α_scan1_). We performed a detailed analysis to determine the optimal regime for whole-brain imaging, the results of which are summarized in Fig. S1–S4.

### Variability and bias evaluation + SAR considerations

We examined the variability and bias of the method in the range of flip angles relevant for brain imaging. To do so, noise was added to the simulated signal and the variability and bias of the method were examined as a function of T_2_ and α. The noise in the simulations was calibrated so the resulting synthetic signal to noise ratio (SNR) matched the measured SNR in agar tubes for the same α and T_2_, where the agar T_2_ was in a range matching white matter (WM) and gray matter (GM) at 7T^18^. The signal dependence on flip angle and phase increment showed that the phase of the signal is high for low φ_inc_ (φ_inc_ < 10°) (Fig. S1). It can be seen that the combination (φ_inc1_ = 3°_,_ α_scan1_) and (φ_inc2_ = 1.5°_,_ α_scan2_ = 2α_scan1_) provides a lower variability (i.e., lower std(T_2_^est.^)) and a smaller bias (i.e., lower |ave(T_2_^est.^) − T_2_^true^|) for a larger range of flip angles (Fig. S2). We also examined three criteria (Fig. S3): the average estimation variability for 30 < T_2_ < 50 ms and 5° < α < 17°, and both the minimal and maximal flip angles that provide std(T_2_^est.^) < 5 ms. The result of a combined minimization of the three criteria (shown in Fig. S3d) is a pair of scans with (φ_inc1_ = 3°_,_ α_scan1_) and (φ_inc2_ = 1.5°_,_ α_scan2_ = 2α_scan1_) that provides a good combination of the lowest average std(T_2_^est.^) and supports a flip angle range of 3.7–35° (in which std(T_2_^est.^) < 5 ms).

Figure S4 shows three additional aspects that were included to establish the final configuration, including the repetition time (TR), the R_FA_ in a realistic experiment and reduction of the cerebrospinal fluid (CSF) signal. Although the combination (φ_inc1_ = 3°_,_ α_scan1_) and (φ_inc2_ = 1°_,_ α_scan2_ = 2α_scan1_) provides a better flip angle range (2.4–35°), in practice, φ_inc1_ = 1° generates a high CSF signal. This can result in an extra signal and a residual artifact in the proximity of the ventricles. To reduce the CSF signal’s effect, it was found worthwhile to use φ_inc2_ = 1.5° (Fig. S4a). As the change in relative variability as a function of TR (Fig. S4b) is insignificant, the choice of TR can be made by balancing between SAR limitations, on the one hand, and scan duration, on the other hand. A TR of 10 ms provided a practical tradeoff. Our examination of the effect of the R_FA_ on the flip angle range showed that the higher the R_FA_, the better (Fig. S4c). However, to keep SAR within the “Normal” level, it was found that R_FA_ in the range of 1.6–2 (with TR = 10 ms) provides a suitable flip angle range. In case of adopting “First level” SAR limit, one can increase the range of the flip angles.

### Global phase corrections

In practice, the phase ($\angle S$) of the signal S at a voxel is comprised of the steady-state phase θ(α, T_2_, T_1_) plus a global phase θ_0_. The global phase θ_0_ arises from several factors, with a dominant contribution from B_0._ It can be eliminated by repeating the scan twice, once with + φ_inc_ and once with -φ_inc,_ and setting θ(α, T_2_, T_1_) = $\angle \left({S}_{{+\varphi }_{inc}}\cdot conj\left({S}_{{-\varphi }_{inc}}\right)\right)/2$ (as was shown in Ref.^27^). The implemented acquisition thus includes four scans: the two scans (φ_inc1,_ α_scan1_) and (φ_inc2,_ α_scan2_) and their repetition with a negative phase increment to remove θ_0_. Calculating the θ_1_ and θ_2_ in this method does not result in phase wrapping, since after the global phase removal, the signals’ phase is in the range of 0° to ~ 50°.

### Estimation algorithm

The actual estimation algorithm included two main steps, per voxel, namely the removal of the global phase (θ_0_) and an estimation of T_2_ and α from (θ_1_,θ_2_) using linear interpolation. An additional step was established for low flip angles because low flip angles result in (θ_1_,θ_2_) measurement pairs close to the edges of the “balloon” (Fig. 2b), a region where interpolation is an ill-posed problem. Low flip angles are relevant for whole-brain imaging because despite the flip angle of the first scan being set to α_scan1_ = 15° , the actual whole-brain RF field distribution results in a flip angle in the range of ~ 4° to 22° (even reaching below 4° for some regions, see a representing distribution in Fig. 1). Brain regions where very low flip angles (~ 4–6°) are typically reached are the cerebellum, midbrain, and brainstem, as well as some regions in the temporal lobe. The added step to handle low flip angles takes advantage of two aspects: i) that α changes slowly in space, and ii) that for small flip angles (α < 20°) the phase $\theta$ is linear with T_2_, and that the slope itself is linear with the flip angle α. Detailed description of this step are in the “Materials and methods” section. Figure S5 shows the improvement attained using the second step for the low flip angles. Additional steps were also performed to improve the estimation for the expected low values of (θ_1_,θ_2_), which, due to noise, results in negative values (see “Materials and methods”).

### T_1_ corrections

As mentioned, phase dependence on T_1_ is small, but it can account for ~ 15% of the final T_2_ estimation. To reduce the error due to T_1_ in human imaging voxels were classified as either “high” or “low” T_1_ by empirically thresholding $\left|{S}_{{\alpha }_{\text{scan2}}}\right|/\left|{S}_{{\alpha }_{\text{scan1}}}\right|$. Separate maps—T_2_(θ_1_, θ_2_) and α(θ_1_, θ_2_)—were used for each classification, based on T_1_ = 1 s (representing WM) and T_1_ = 2 s (the rest). With this correction, the error was further reduced (shown in Fig. S6). A detailed description of the algorithm is provided in the “Materials and methods” section.

## Results

**Figure 3:**
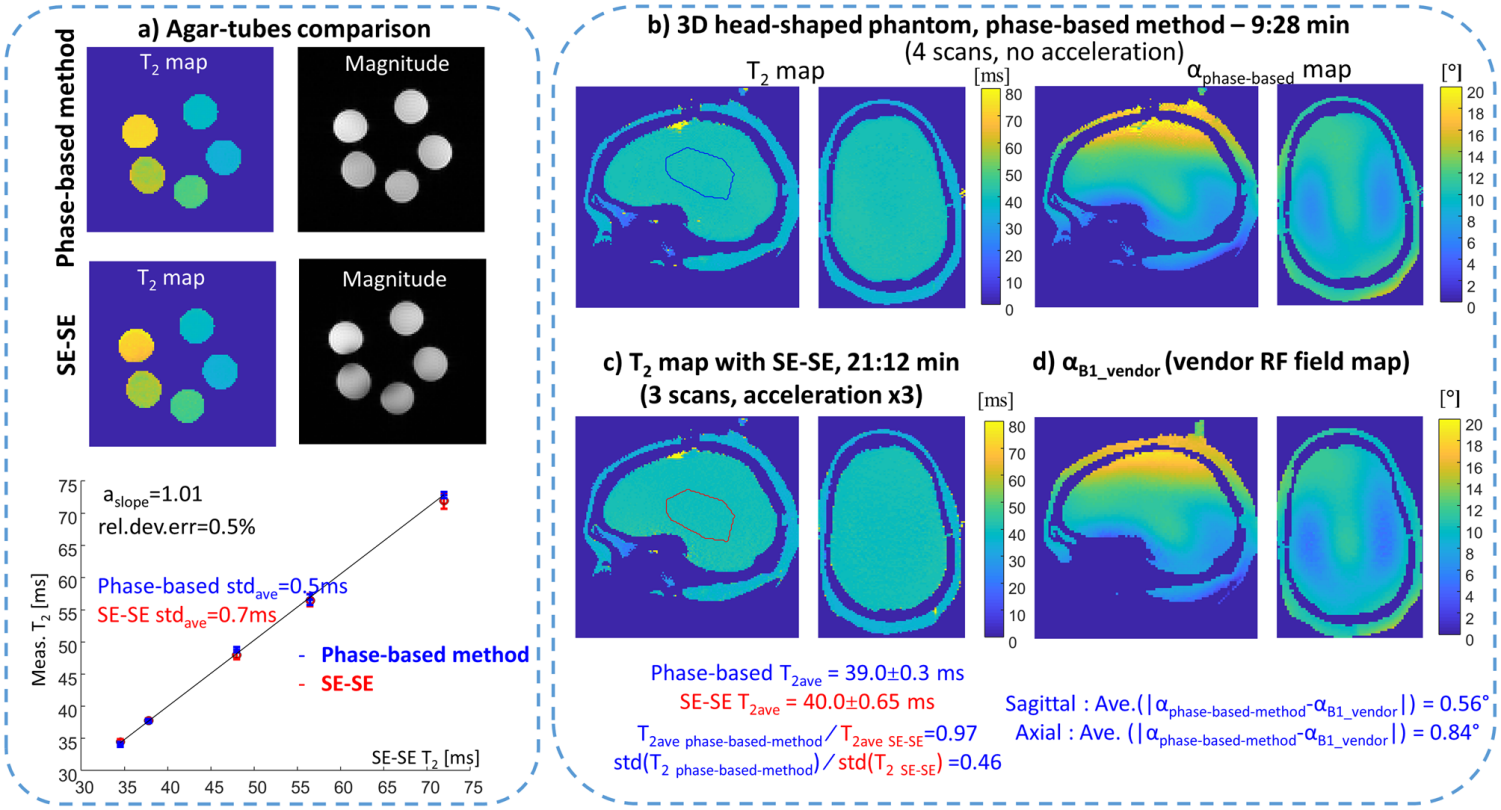
Assessment of estimation bias and variability in phantoms. (**a**) Comparison of the T_2_ obtained with the phase-based method and SE–SE in agar tubes. Top—a central slice of the T_2_ maps and magnitude images. Bottom—estimated T_2_ for each tube as a function of T_2_ with SE–SE; a_slope_ = 1.01, relative deviation error = 0.5%. The average standard deviation was 0.5 ms for the phase-based method, and 0.7 ms for SE–SE. (**b**–**d**) Comparisons using a 3D-head-shaped brain-like phantom. (**b**) T_2_ and α maps estimated by the phase-based method. (**c**) T_2_ map estimated with SE–SE. And (**d**) α map estimated using the vendor’s RF field mapping scan. Two main cross-sections are shown for all cases, Sagittal and Axial. For comparison, the average T_2_ and standard deviation was calculated in the same region of interest (marked by a blue contour for the phase-based method and a red contour for SE–SE). The average deviation between the α maps of the phase-based method and of the vendor’s RF mapping was calculated to be 0.56° for the Sagittal plane and 0.84° for the Axial plane.

To examine the estimation bias and estimation variability we conducted two imaging experiments with phantoms, one with tubes filled with agarose suspension, the other with a 3D head-shaped phantom. In the first experiment (Fig. 3a), the variability was × 1.4 smaller than with SE–SE (0.5 ms compared to 0.7 ms). The a_slope_ and the relative deviation error (see Eq. 1) calculated between the T_2_ from this method and the T_2_ from SE–SE were 1.01 and 0.5%. Thus, the phase-based method provides a small bias and a lower variability compared to SE–SE, while the scan duration is × 2.3 faster.

In the second experiment, a specially designed 3D head-shaped brain-like phantom was used to examine the capability to cope with an RF field distribution similar to that in the brain. The “brain” had a uniform T_2_, which helped to separate the two parameters we sought to estimate, α and T2. Our results show low variability in T_2_ (std(T_2 phase-based-method_ )/std(T_2 SE–SE_) = 0.46) and an RF field map estimation with little bias (a 4% average deviation from the map acquired with the vendor’s pulse sequence), see Fig. 3b. Even low flip angles, in the ill-posed area of the “balloon”, were well determined using the implemented estimation algorithm (Fig. S8).

**Figure 4:**
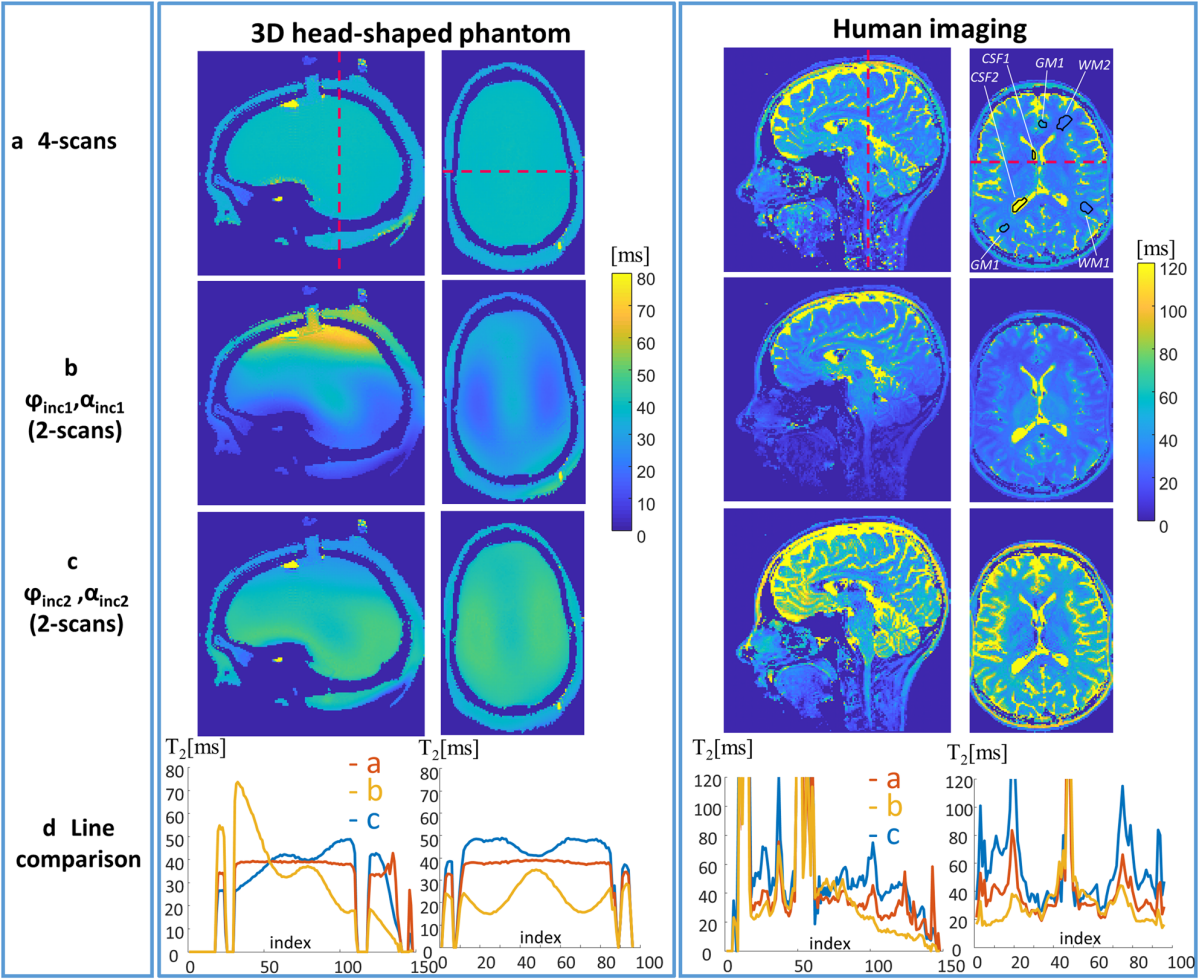
Comparison of T_2_ maps extracted with (**a**) 4-scans, (**b**) single pair with (φ_inc1,_ α_scan1_) and (**c**) single pair with (φ_inc2,_ α_scan2_). (**d**) For each case a plot for a line shown in the Sagittal and Axial scans. The images show 3D-head shaped phantom (left) and human imaging (right). The human axial plane image in (**a**) shows the regions that were examined and summarized in Table 1.

The contribution of the B_1_ correction to the T_2_ estimate can be seen in Fig. 4. It compares T_2_ maps extracted from a set of four scans (two pairs) to T_2_ maps extracted from a single pair—as in Ref.^27^—using either of the pairs (either pair 1: φ_inc1_ = 3° and φ_inc1_ = − 3°, with α_scan1_; or pair 2: φ_inc2_ = 1.5° and φ_inc2_ = − 1.5°, with α_scan2_). It can be seen that for both phase increments the RF field inhomogeneity results in either underestimated or overestimated T_2_ values, depending on the actual flip angle in each voxel (see Fig. 2a for phase dependence on flip angle). The 4-scans result, which combines both phase increments, provides a uniform T_2_ map of the “brain” tissue in the 3D-head shaped phantom, as expected by the design.

**Table 1:** Estimated T_2_ in sample regions of white matter, grey matter and CSF (see Fig. 4).

	Single pair (φ_inc1,_ α_scan1_)	Single pair (φ_inc2,_ α_scan2_)	4-scans	From Ref.^18^
WM1	19.00 ± 0.86	40.02 ± 2.34	28.83 ± 1.47	33.7 ± 0.7
WM2	16.28 ± 1.21	37.84 ± 4.04	26.74 ± 2.49	
GM1	30.02 ± 5.77	60.67 ± 14.52	45.05 ± 10.23	49.2 ± 3.8
GM2	25.37 ± 3.10	59.05 ± 14.97	41.95 ± 9.52	
CSF1	341.93 ± 105.73	447.95 ± 31.76	422.75 ± 33.30	
CSF2	280.25 ± 3.10	436.36 ± 14.64	408.35 ± 15.73	

Figure 4 also shows the estimated T_2_ maps, for human imaging, based on either 4-scans or a single scan-pair. Although more challenging to observe, due to the heterogeneous T_2_ distribution in the brain and to the very high T_2_ values in the CSF regions, it can also be seen that T_2_, estimated from a single pair, is either underestimated or overestimated compared to 4-scans. This can be observed, for example, in regions such as the cerebellum and the temporal lobes. Table 1 summarizes the results by giving sample T_2_ values in white matter, grey matter and CSF. For each tissue 2 sampled regions were chosen as shown in Fig. 4—WM1 and WM2 in white matter tissue, GM1 and GM2 in the grey matter tissue and CSF1, CSF2 in the CSF. The table also shows T_2_ values reported in Ref. ^18^. Note: the CSF values are underestimated with the current method, as further elaborated in the Discussion section.

**Figure 5:**
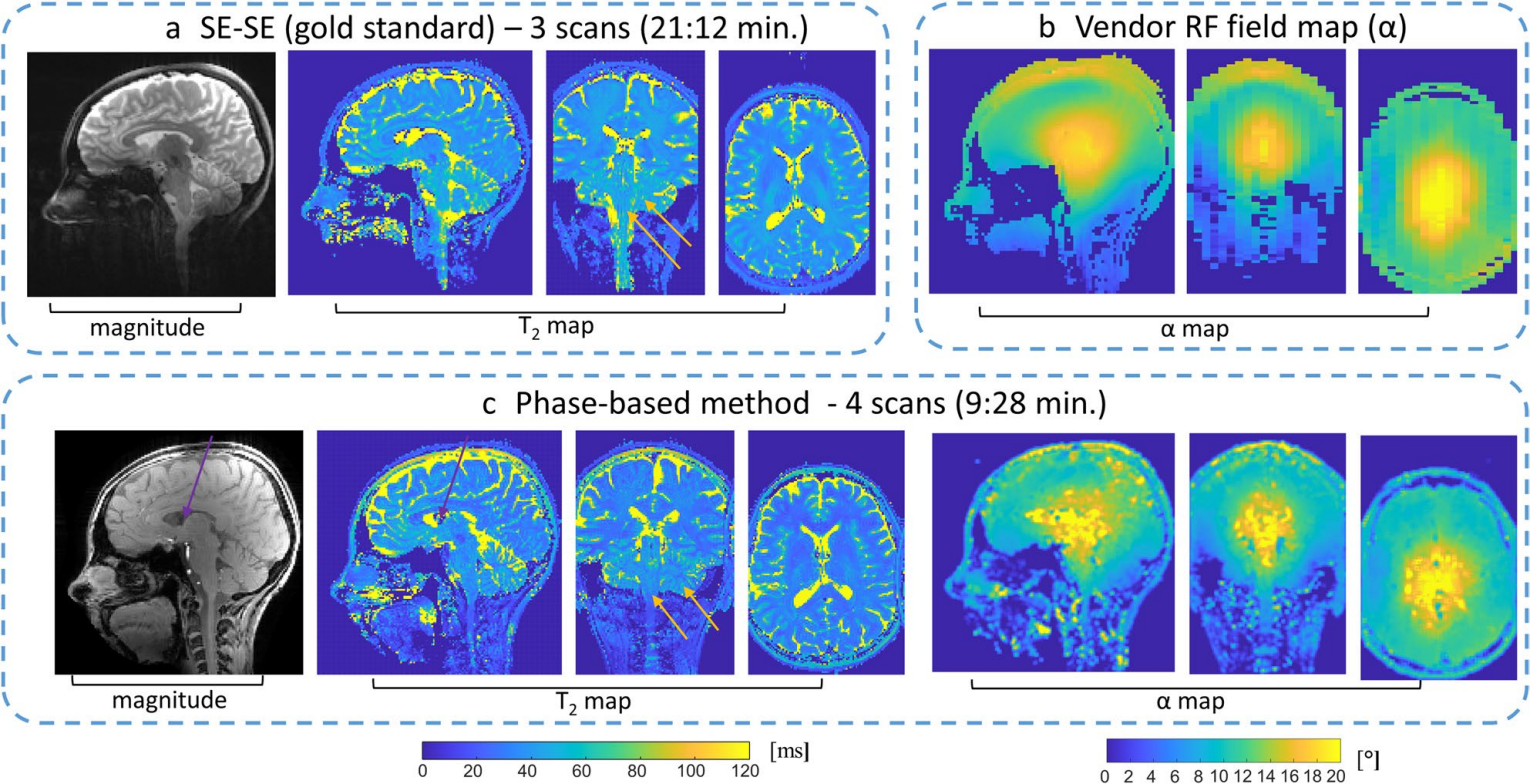
Human imaging—T_2_ from the phase-based method or SE–SE, and α from the phase-based method or the vendor’s scan. (**a**) SE–SE Sagittal magnitude image at TE = 30 ms and the estimated T_2_ maps in three main cross-sections. (**b**) An α map using the vendor’s pulse sequence. (**c**) Sagittal magnitude image with φ_inc_ = 3 and α = 15°, as well as the estimated T_2_ and α maps in three main cross-sections. α map shown here was smoothed by a 3 × 3 filter to reduce the effect of local CSF signal. Orange arrows point to the cerebellum and brainstem regions suffering from low flip angles due to B_1_ inhomogeneity; their inner structure is much more pronounced—and clearly visible—in the phase-based T_2_ images. Purple arrows point to a region in the CSF that resulted in a low magnitude signal.

**Figure 6:**
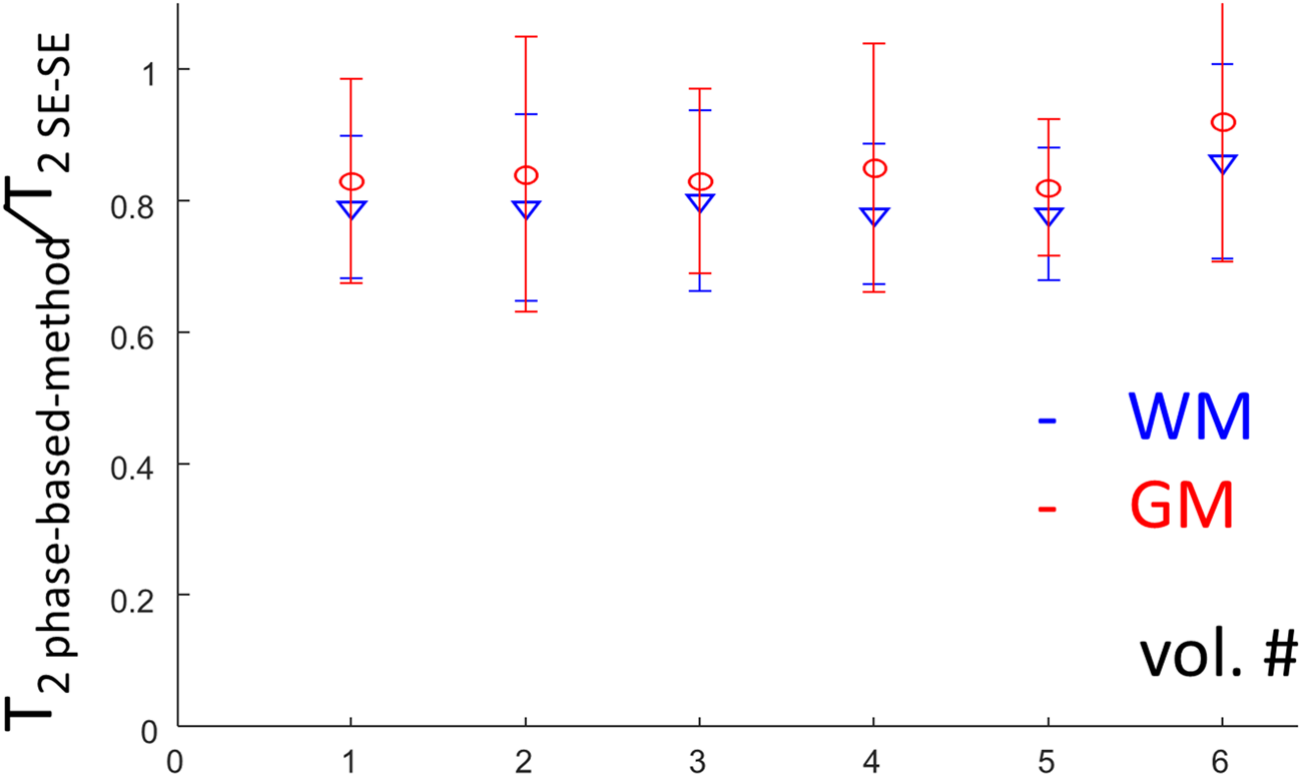
Comparison of T_2_ estimation between the phase-based method and SE–SE. The plot shows the ratio T_2 phase-based-method_/T_2 SE–SE_ per volunteer, both for WM and for GM. The error bars depict the relative deviation error [see Eq. (1)].

Continuing with human imaging, Fig. 5 compares the phase-based method with 1.5 mm isotropic voxels to the gold standard SE–SE, for a T_2_ mapping comparison, and to the vendor RF mapping, for an RF field mapping comparison. The α map in Fig. 5c was smoothed by 3 × 3 filter to reduce the effect of local CSF signals (see Fig. S13 for original high resolution B_1_ map). The RF field map extracted with the phase-based approach shows a distribution similar to the separately acquired vendor map with, however, noticeable deviations in the ventricles, as well as in some of the CSF region. The ratio of the T_2_ values and the relative deviation error between the phase-based method and SE–SE is shown in Fig. 6, for the different volunteers. Over all volunteers the T_2_ ratio (T_2 phase-based-method_/T_2 SE–SE_) and relative deviation error are 0.80 and 15.45% for WM, and 0.85 and 19.76% for GM (detailed description is in Supplementary Information S4).

**Figure 7:**
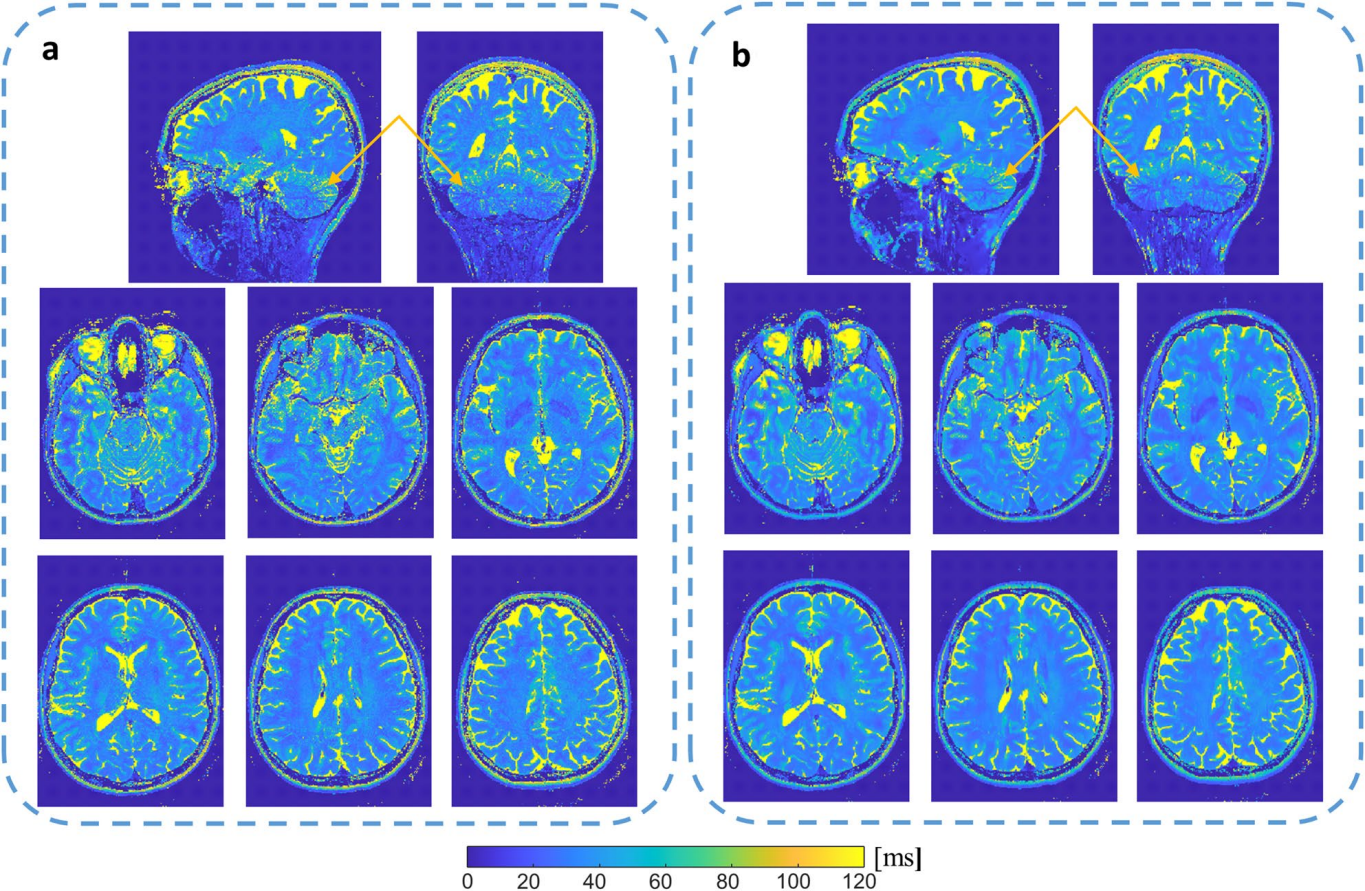
Human whole-brain T_2_ maps with a 0.85 mm isotropic voxel. (**a**) without denoising, (**b**) with denoising, based on DnCNN model for Gaussian noise removal. Arrows point to the cerebellum region, which especially benefits from denoising. Top row, Sagittal and Coronal planes. Bottom two rows, six slices of the Axial plane, at 10 mm intervals.

Finally, high-resolution whole-brain T_2_ mapping was performed with the phase-based method, with 1 mm and 0.85 mm isotropic voxels. To acquire whole-brain high-resolution images, × 5.11 acceleration was used—combining elliptic sampling and × 2 acceleration in both phase encoding directions. Each of the four scans with 1 mm resolution was 1:13 min giving a total scan time of 4:52 min. For 0.85 mm each scan was 1:42 min long and the total scan time was 6:48 min. Figure 7 shows the estimated T_2_ maps for the 0.85 mm scan (Fig. S12 shows the 1 mm resolution images). To provide even higher robustness following the reduced SNR of the high-resolution datasets, we also incorporated denoising based on a DnCNN deep-learning network^33^ (provided in MATLAB, The Mathworks, Natick MA, for Gaussian noise removal). This entailed denoising of θ_1_ and θ_2_ before the estimation of T_2_. The denoising greatly improved the observed details of the cerebellum structure, a region with especially low flip angles (Fig. 7b).

## Discussion

The expected rewards of pushing the limits and moving to 7 T MRI are increased spatial resolution and shorter scan durations. Both these features are essential for clinical and research imaging, all the more so for quantitative methods. However, scanning at 7 T also poses new challenges, including high power deposition and severe RF field inhomogeneity. The extended phase-based method shown here delivers high-resolution brain T_2_ imaging while overcoming the above challenges. This is achieved by relying on a modified 3D SGRE sequence, using the phase of the signal to encode the T_2_ dependence. The 3D SGRE images are also highly robust to B_0_ inhomogeneity. This can be seen in the magnitude images of both the phantom example (Fig. 3a) and the human images (Fig. 5). The SE–SE is more distorted both at the edges of the agar tubes and near the nasal areas in the human images. The B_0_-dependent phase is reliably canceled out by the two scans with opposite phase increments (φ_inc_) of the RF pulse train. However, shifts in the global phase between scans may occur, which will require corrections. Similarly, the scans may be sensitive to movements, which will affect the phase. Incorporating a second echo acquisition could be used to correct for both the phase shifts and motion^34^. Aiming to shorten the total scan duration, one can also consider estimation of the global phase from a single pair, thus reducing the number of scans to three. However, in this case careful analysis and phase unwrapping will be required in the third, non-paired, scan. In this case, phase unwrapping can be especially challenging in regions with short T_2_^*^, where B_0_ changes rapidly, resulting in high local changes in the background phase. Very short T_2_^*^ may also affect T_2_ estimation due to limited SNR in such regions.

The current implementation used a non-selective hard pulse for the 3D acquisition. Although this works well for whole brain acquisition as in this study, in other cases it can be a limitation. For faster acquisition and to limit potential aliasing, the use of slab-selective pulses is beneficial. Figure S9 shows that as long as the slab is thick enough, compared to the slice thickness, the estimated T_2_ is correctly estimated. However, for a single slice-selective acquisition, the simulation by which the T_2_ and RF field maps are estimated must also account for the slice profile. This was already demonstrated in other T_2_ mapping methods such as balanced SSFP^19^.

Another sensitivity of the method that requires discussion is the sensitivity to movement and potential inaccuracy in the RF pulse phase. Although we did not observe noticeable movement in our human scanning, a simulation to examine these vulnerabilities was performed (see Supplementary Information, Section S5). The movement was simulated assuming a constant velocity during the scan, which will result in an additional parabolic phase term accumulated during the scan. Examining the error due to potential head movement of 1–2 voxels during the scan, it resulted in a small error, less than 1% for a movement of up to 5 mm/min. However, for large movement within a voxel, such as due to flow, the error of the estimated T_2_ can be significant; reaching 20%, for a velocity of 0.5 mm/s.

Two simulations were also performed to analyze possible hardware inaccuracies: (i) a constant error in the actual RF-phase increment, (ii) a randomly distributed error in the actual phase of the RF pulse. In the first case, a constant error of 0.1° resulted in < 4% error, In the second, a randomly distributed error with σ = 0.2° resulted in a negligible error with standard deviation of 0.07 ms in the estimated T_2_. It is also important to note that the estimation of the T_2_ in the CSF and other tissues with high T_2_ values (> 0.5 s) is challenging with this method, since the signal’s phase curve slowly converges for T_2_ > 100 ms (see Fig. 2a) and so the T_2_ contours in the (θ_1_,θ_2_) space grow denser with T_2_ (see Fig. 2b). In addition, local intensity drops in CSF voxels, resulting in low SNR voxels, can occur due to fluid movement (purple arrows in Fig. 5 point to such area), thus further limiting T_2_ estimation of CSF.

The important advantage of the phase-based approach for T_2_ mapping is its whole-brain coverage ability. The method shows robust results in the brainstem region and even in parts of the spinal cord (see Fig. 5). These results are achieved without the need for additional hardware to reduce the RF field inhomogeneity, such as dielectric pads or multi-channel transmit coil. Naturally, the method can also benefit from a dielectric pad or multi-channel transmit coils to improve the SNR, especially in regions with low flip angles. The current configuration (φ_inc1_, φ_inc2_, α_scan1_, R_FA_, and TR) was designed for the RF field distribution in the brain, and was shown to robustly extract the RF field distribution in the 3D head-shaped phantom (which has a slightly larger RF field inhomogeneity than in vivo). If another region will be of interest, the configuration—the RF pulse phase increments and the scan flip angles—can be adapted accordingly.

It is worth noting that θ_1_ on its own, calculated from the first pair of scans (with φ_inc1_ = ± 3°), achieves a “T_2_ weighted” image (see Fig. S10 for the 0.85 mm case), unlike the magnitude of these scans. θ_1_, however, suffers from pronounced RF field inhomogeneity, which is removed by using two sets of scans (giving θ_1_ and θ_2_), as was implemented here, allowing the generation of T_2_ maps.

In our study, the estimation algorithm is based on an interpolation procedure, where the simulated data serves as the ground-truth. This method is similar to the dictionary-based approach in MRF, but is based on two measurement points (θ_1_, θ_2_) that allow us to represent the parameters of interest, T_2_ and α, in the (θ_1_, θ_2_) 2D space. This offers the advantage of mapping the T_2_ of interest by a simple linear interpolation. An improvement in the estimation algorithm was implemented in the low flip angles’ range, which extended the viable flip angles (Figs. S5 and S8). In this study, we demonstrated the low variability and small bias of the estimations in both simulations and phantom experiments. In the phantom experiment with agar tubes, the method provides T_2_ estimation with low variability—a × 3.2 (1.4 × 2.3) lower variability-to-scan-time factor than that of SE–SE. The T_2_ values were estimated by the phase-based method with a small bias (a_slope_ = 1.01 and relative deviation error of 0.5% compared to SE–SE).

However, the in-vivo T_2_ ratio of the phase-based method to SE–SE was 0.79 ± 0.16 for WM and 0.86 ± 0.19 for GM. Similarly, there is a ratio of × 0.82 and × 0.88 between the reported values with 4-scans in Table 1 to the values in Ref.^18^. This result is also similar to the results in ﻿Ref.^22,34^. Possible reasons for the different ratios found for WM and GM are a partial volume of GM and CSF as well as deviations due to T_1_. Although T_1_ has a small impact on the phase of the signal and its effect was reduced in our implementation. The ~ 0.8 ratio between the T_2_ estimated by the phase-based approach and by SE–SE could arise for several reasons, among which are a contribution due to exchange and magnetization transfer^35^, diffusion^36^, and different contributions of the fast and slow T_2_ components to the two methods^37,38^. For the magnetization transfer no discrepancy was observed between the estimated T_2_ values in the agarose tubes, although exchange mechanisms are known to be at work in agarose and therefore produce magnetization transfer effects. However, different effects of exchange in the living tissue can still be a factor contributing to the acquired complex signal of the steady state acquisition. We also examined potential diffusion contributions to the estimated T_2_ by scanning a sample of smoked fish (which had and ADC of ~ 0.6 × 10^−3^ mm^2^/s, similar to white matter) and did not observe a significant effect (not shown). One of the potential factors is the larger contribution of the fast-relaxing species compared to SE–SE, primarily due to much shorter echo times, which was also observed in several previous studies^38^. Thus, although the estimated T_2_ was robustly repeated in the volunteers’ data, the resulting ratio between the phase-based method and SE–SE *in-vivo* still requires further analysis.

The fast high-resolution T_2_ maps of the whole brain that were acquired—1 mm isotropic in 4:52 min and 0.85 mm isotropic in 6:49 min—offer a significant clinical gain. Further acceleration of the method should be possible. One option is to reduce the TR, however, this will require switching the SAR monitoring to the less restrictive “First” level. For this, the effect of the TR on the variability of the estimation was examined (see Fig. S4) and showed that shorter TR result in similar estimation variability. In addition, acceleration methods, tuned to the 3D SGRE acquisition and employing the Compressed Sensing technique, can achieve even higher acceleration factors. We also demonstrated the option of employing denoising based on deep-learning techniques that is trained to remove Gaussian noise. This further improves the quality of the images and can be used to further accelerate the scan.

Overall, the extension of the phase-based steady-state method to estimate both T_2_ and RF field map, demonstrated in this work, provides a fast and high-resolution acquisition method for quantitative T_2_ mapping of the whole brain at 7 T acquired with a single-channel transmit coil. Standardized high-resolution methods are imperative for 7 T MRI to advance multi-site studies and promote personalized medicine.

## Materials and methods

### Bloch simulations

1D single voxel simulations based on the Bloch equations were performed with a custom MATLAB (The Mathworks, Natick MA) code^39^ to examine the signal in steady state. The simulations included an excitation pulse, an acquisition and a net total spoiler (including the area of the acquisition) of 3/Δx (Δx the 1D voxel size). The number of initial repetitions to reach steady-state (“dummy scans”) was set to 500, which was verified to provide reliable steady states. Following the dummy scans, a single acquisition was simulated. The simulation was repeated over a grid of flip angles and T_2_ values, for different values of T_1_, φ_inc_, and TR. The grid covered T_2_ from 0 to 200 ms with a resolution of 4 ms, and flip angles from 0° to 70° with a resolution of 1°. The resulting θ(T_2_, α) map was interpolated prior to its use in the estimation algorithm with 1 ms in T_2_ and 0.1° in alpha, generating θ_1_(T_2_, α) and θ_2_(T_2_, α) for relevant φ_inc_ and R_FA_ factors.

### Estimation algorithm

The estimation algorithm included the following steps:

#### Preparatory step #0.1: global phase removal

In practice, the phase ($\angle S$) of the signal S at a voxel is comprised of the steady-state phase θ(α, T_2_, T_1_) plus a global phase θ_0_. The global phase θ_0_ arises from several factors, with a dominant contribution from B_0._ It can be eliminated by repeating the scan twice, once with + φ_inc_ and once with -φ_inc,_ and setting θ(α,T_2_,T_1_)=$\angle \left({S}_{{+\varphi }_{inc}}\cdot conj\left({S}_{{-\varphi }_{inc}}\right)\right)/2$ (as was shown in Ref.﻿^27^). The implemented acquisition thus includes four scans: the two scans (φ_inc1,_ α_scan1_) and (φ_inc2,_ α_scan2_), and their repetition with a negative phase increment to remove θ_0_.

#### Preparatory step #0.2 (optional): denoising

For high-resolution human imaging, a denoising procedure based on a DnCNN deep-learning network^33^ (provided in MATLAB 2021a, for Gaussian noise removal) was incorporated. The denoising procedure was implemented on the measured θ1, θ2 with the command denoised_θ1 = denoiseImage(θ1, net), where the net was set by the command net = denoisingNetwork('dncnn').

#### Estimation step #1: T_2_ and α estimation by interpolation

First, using Matlab’s scatteredInterpolant(), we generated two interpolants, T_2_(θ_1_, θ_2_) and α(θ_1_, θ_2_), which map (θ_1_, θ_2_) to the desired quantities T_2_ and α. These interpolants were then used to estimate T_2_ and α from any (θ_1_, θ_2_) pair, at each voxel.

As mentioned, phase dependence on T_1_ is small, but it can account for ~ 15% of the final T_2_ estimation. Thus, in human imaging, to reduce the error due to T_1_, voxels were classified as either “high” or “low” T_1_ by empirically thresholding $\left|{S}_{{\alpha }_{\text{scan2}}}\right|/\left|{S}_{{\alpha }_{\text{scan1}}}\right|$. Separate maps—T_2_(θ_1_, θ_2_) and α(θ_1_, θ_2_)—were used for each classification, based on T_1_ = 1 s (representing white matter—WM) and T_1_ = 2 s (the rest). With this correction, the error was further reduced (see simulation results in Fig. S6).

#### Estimation step #2: T_2_ estimation update for low flip angles

First, the flip angles α found in the previous step were smoothed, generating α_smoothed_. For low flip angle voxels with α_smoothed_ < 4.5°, the flip angles were temporarily set to α_temp_ = 4.5°, and the matching temporary T_2_ quantities, T_2-temp_, were found by interpolation—using α_temp_ and θ_2_ (the phase from the scan using the higher flip angle, αscan_2_ = RFA∙αscan_1_). The final T_2_ was found through the linear connection T_2_ = (α_temp_/α_smoothed_)∙T_2-temp_.

#### Estimation step #3: handling of negative θ_1_ or θ_2_

For θ_1_ < 0 (and θ_2_ > 0), the θ_2_ from step #1 together with α_smoothed_ from step #2 were used to estimate T_2_; using the above simulated θ_2_(T_2_,α) for the known α. Similarly, for θ_2_ < 0 (and θ_1_ > 0), θ_1_ and α_smoothed_ were used to estimate T_2_.

Validation of the estimation algorithm was performed by generating N = 100 noisy repetitions of each point in the simulated datasets of θ_1_(T_2_,α) and θ_2_(T_2_,α). This was done using a fixed noise which resulted in the SNR varying with T_2_ and α, depending on the intensity at each point. The noise was fixed to produce an SNR of 180 for the simulated data at T_2_ = 38 ms and α = 13°; resembling the SNR in the human images acquired with 1.5 mm resolution. The SNR was set as an average SNR over the two signals |S_1_| and |S_2_|. To validate the simulations, the standard deviation of T_2_ was compared to a measured one in an agar-tubes experiment, both with the same SNR. For this validation two agar-tubes were used—with T_2_ values of T_2_ = 34 ms and T_2_ = 38 ms, representing WM and GM at 7 T. The flip angle distribution in this experiment was uniform (α = 13°). The measured and simulated SNR was 298, resulting in a T_2_ standard deviation of 0.36 ms in the measurement and 0.32 ms in the simulation, providing comparable results. The variability and bias of the method, under the simulated noise, were examined as a function of T_2_, α, φ_inc_, TR and R_FA_.

### Pulse sequence considerations

The sequence is based on a Siemens 3D GRE sequence that was modified to enable control over both the φ_inc_ and the gradient spoiler moment. The RF pulse we used was a hard pulse.

An important aspect to consider is the gradient spoiler moment intensity and its effect on the T_2_ estimation, as well as on image artifacts (in the form of residual signals from spurious echoes). A set of scans was performed to examine the spoiler effect. The gradient spoiler moment needs to provide complete dephasing inside a voxel, which defines a preferable gradient moment size to be $\succsim$ 1/Δr (Δr = √(Δx^2^ + Δy^2^ + Δz^2^)). We found it useful to add a parameter to the pulse sequence that directly controls the net gradient spoiler moment (after all previous gradients had been rephased). The net spoiler was set to be equally distributed in all three directions, which was found useful in reducing artifacts. However, our experiments also showed that the gradient moment affected the measured phase, and thus the estimated T_2_. Figure S7a shows this dependence. Phantom experiments were used to calibrate the gradient spoiler moment to provide the T_2_ estimate closest to that from SE–SE. Accordingly, the gradient moment was set in all experiments to 0.015 [mT/m∙sec] in each direction. This moment is expected to provide dephasing for Δr $\succsim$ 0.9 mm. As shown in Fig. S7b, under this moment, the estimated T_2_ did not change for the voxel sizes tested.

### MRI scanning

All scans in this study were performed on a 7 T MRI system (MAGNETOM Terra, Siemens Healthcare, Erlangen) using a commercial 1Tx/32Rx head coil (Nova Medical, Wilmington, MA).

When comparing the results of the phase-based method to SE–SE, inside a region, the relative deviation error from the fit was calculated as

1 $$\text{rel. dev. err}=100\cdot \frac{\sum_{i=1}^{N}\left|\left({T}_{2}^{\text{(phase-based-method)}}-{a}_{slope}{T}_{2}^{\text{(SE-SE)}}\right)\right|}{N}/{\text{ave}}\left({a}_{slope}{T}_{2}^{\text{(SE-SE)}}\right)$$

where *a*_*slope*_ is the slope found for each fit, and N is the number of voxels in the comparison.

### Phantom imaging

Five tubes with agar concentrations of 1.5, 2, 2.5, 3 and 3.5% were used to compare the phase-based T_2_ estimation to the gold standard SE–SE, using three TE values (10, 30 and 50 ms). A 3D head-shaped phantom that was designed to model the RF field distribution in the brain was used to examine the T_2_ and RF field estimation. This phantom was originally designed to include three sub-compartments^30^, suitable for mimicking brain, muscle and lipid tissues. However, the version used in this study was filled with two “tissue” types: the inner compartment mimicked the “brain” and the outer one, “muscle” (the planned lipid layer was also filled with “muscle”). Both compartments contained 0.1 mM gadopentetate dimeglumine (GdDTPA), for a T_1_ close to that of human white matter, and consisted of an agarose suspension of 2.5% and 3% for the “brain” and “muscle” compartments, respectively. NaCl (5.5 gr/L) was used to achieve an *in-vivo*-like RF field distribution. For details, see Ref.^30^.

α maps from the phase-based method were compared to the equivalent α maps generated by the vendor. As the RF field maps provided by the vendor are scaled to 90°, they were rescaled to the α_scan_ of the phase-based method, before comparison. The average deviation between the α maps by the phase-based method and by the vendor were calculated in two main planes (Sagittal and Axial).

The common scan parameters for the phase-based method and SE–SE used in the agar-tube experiments in Fig. 3a) were: FOV 200 × 200 × 104 mm^3^, resolution 1.1 × 1.1 × 2 mm^3^, acquired matrix size 176 × 176 × 52. The phase-based method specific parameters were (φ_inc1_ = 3°, α_1_ = 15°) and (φ_inc2_ = 1.5°, α_2_ = 30°), TR/TE 10/2.2 ms, using 4 scans with a total scan duration of 6:06 min. The specific scan parameters for SE–SE were: TR—6500 ms, 3 scans with TE = 10,30,50 ms, × 3 in-plane acceleration, with a total scan duration of 19:04 min. The T_2_ and α maps were estimated based on Bloch simulation with T_1_ = 2 s.

The common scan parameters for the phase-based method and SE–SE that were used for the 3D head-shaped phantom in Fig. 3b): FOV 220 × 220 × 144 mm^3^, isotropic resolution of 1.5 mm, bandwidth per pixel 400 Hz. The phase-based method specific parameters were acquired matrix size 150 × 148 × 96, (φ_inc_ = 3, α = 15°) and (φ_inc_ = 1.5, α = 30°), TR/TE 10/2.1 ms, using 4 scans with a total scan duration of 9:28 min. The specific scan parameters for SE–SE (Fig. 3c) were: acquired matrix size 144 × 144 × 96, TR—6500 ms, 3 scans with TE = 10, 30, 50 ms, × 3 acceleration, with a total scan duration of 21:12 min. The vendor RF field map scan parameters (Fig. 3d): FOV 220 × 220 × 144 mm^3^, resolution 2.3 × 2.3 × 4 mm. The T_2_ and α maps were estimated based on Bloch simulation with T_1_ = 1.5 s (based on estimated T_1_ of the “brain” tissue).

### Human imaging

All methods were carried out in accordance with the Weizmann Institute of Science guidelines and regulations. This study was approved by the Internal Review Board of the Wolfson Medical Center (Holon, Israel) and all scans were performed after obtaining informed suitable written consents. Human scanning of six volunteers with isotropic 1.5 mm resolution was acquired for the comparison with SE–SE. The comparison was performed after the SE–SE and the phase-based method images were realigned using SPM12 (https://www.fil.ion.ucl.ac.uk/spm/) to ensure there was no movement between the scans.

An additional volunteer was scanned with the phase-based method with 1 mm and 0.85 mm resolution. These scans were acquired with an acceleration of × 5.11—using elliptical sampling and × 2 acceleration in both phase encoding directions. The BART^40^ software was used to reconstruct this dataset.

#### Scan parameters for the phase-based method and SE–SE comparison with isotropic 1.5 mm voxel

Phase-based method: FOV 220 × 220 × 144 mm^3^, acquired matrix size 150 × 148 × 96, bandwidth per pixel 400 Hz, TR/TE 10/2.1 ms, (φ_inc1_ = 3°,α_scan1_ = 15°), (φ_inc1_ = 1.5°, α_scan2_ = 24–26°) (the α_scan2_ varied from 24° to 26° according to the specific volunteer’s 100% “Normal” SAR level), with duration of 4 scans—9:28 min. SE–SE: FOV 220 × 220 × 132 mm^3^, acquired matrix size 144 × 144 × 88, bandwidth per pixel 400 Hz, TR- 6500 ms, TE = 10, 30, 50 ms, using 3 scans with a total scan duration of 21:12 min. Vendor RF field map scan parameters: FOV 220 × 220 × 192 mm^3^, resolution 3 × 3 × 4 mm.

#### Phase-based method 1 mm resolution parameters

FOV 220 × 220 × 160 mm^3^, bandwidth per pixel 400 Hz, TR/TE 10/2.7 ms, (φ_inc1_ = 3°,α_scan1_ = 15°), (φ_inc1_ = 1.5°, α_scan2_ = 25°), duration of 4 scans—4:52 min.

#### Phase-based method 0.85 mm resolution parameters

FOV 220 × 220 × 163 mm^3^, bandwidth per pixel 400 Hz, TR/TE 10/2.7 ms, (φ_inc1_ = 3°,α_scan1_ = 15°), (φ_inc1_ = 1.5°, α_scan2_ = 25°), duration of 4 scans—6:49 min.

## Supplementary Information

Supplementary Information 1.

Supplementary Movie S1.

Supplementary Movie S2.

Supplementary Movie S3.

Supplementary Movie S4.

Supplementary Movie S5.

Supplementary Movie S6.

## Data Availability

All scans collected in this study were performed according to procedures approved by the Internal Review Board of the Wolfson Medical Center (Holon, Israel). Since this protocol was not defined as an open repository, the data is not provided, to provide the ethics and privacy issues of clinical data. The code will be made available via a request to the corresponding author.
