# Supplementary material for: Phase-based fast 3D high-resolution quantitative T2 MRI in 7 T human brain imaging
Source: Sci Rep. 2022 Aug 18;12:14088. doi: 10.1038/s41598-022-17607-z (PMC9388657; doi:10.1038/s41598-022-17607-z)
Supplement: Supplementary file 1 — Supplementary Information 1. [file 41598_2022_17607_MOESM1_ESM.pdf]

Supplementary Information for
Phase-based fast 3D high-resolution quantitative T_2 MRI in 7T human brain imaging

*Amir Seginer[1] and Rita Schmidt[2,3]**

[1]Siemens Healthcare Ltd, Rosh Ha'ayin, Israel

[2]Weizmann Institute of Science, Department of Brain Sciences, Rehovot, Israel

[3]The Azrieli National Institute for Human Brain Imaging and Research, Weizmann Institute of Science

*Rita Schmidt - corresponding author.

Email: rita.schmidt@weizmann.ac.il

S1. Variability and bias analysis of the T_2 estimation

Effect of scan parameters on Variability and bias

Simulations of the steady-state signal (magnitude and phase) based on Bloch equations were performed. Figure S1 shows the magnitude and phase of the signal as a function of φ_{inc} and α for T_2=35 ms, T_1=1 sec, and TR=10 ms. This simulation shows that for small φ_{inc} (φ_{inc}<~10°) the signal decreases with φ_{inc}, while it increases with the flip angle. The short TR was chosen as to balance between scan duration and SAR concerns. Based on these simulations, the φ_{inc} considered in this work were up to 5°;

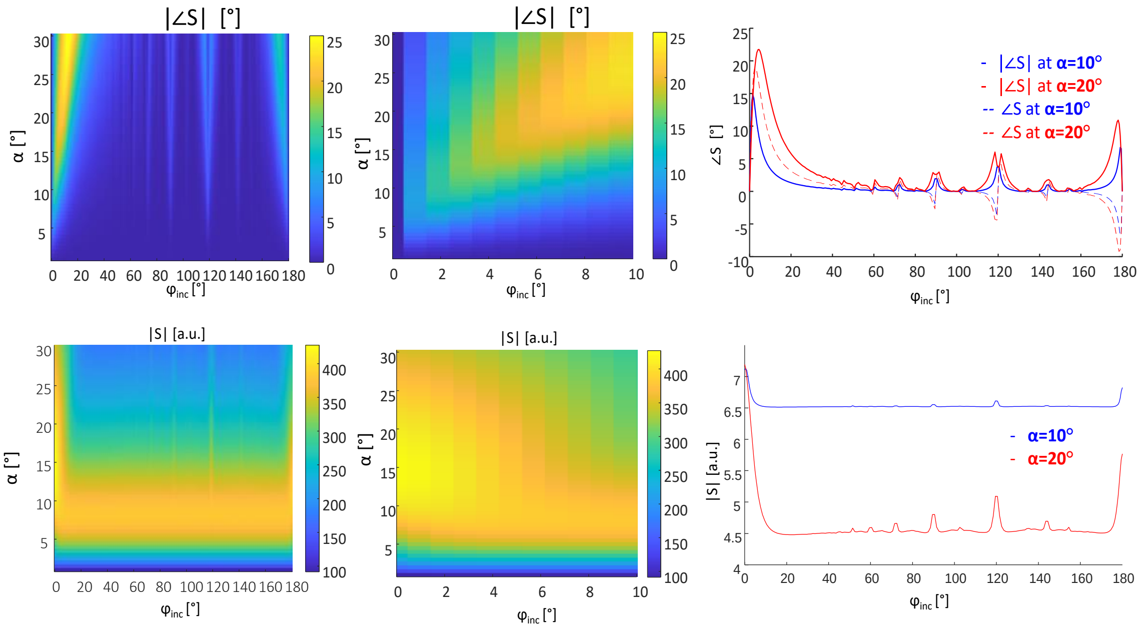

Fig. S1. Steady-state signal as a function of φ_{inc} and α. Simulated for T2=35 ms, T1=1 sec, and TR=10 ms. Magnitude and absolute phase are shown, as they are the main factors towards the attainable contrast-to-noise. From left to right – the phase/magnitude of the signal for the full range of α and φ_{inc}, zoom-in with φ_{inc}<10° and 1D plots for α=10° and 20°.

having high relative signal and high phase values, which provide a low estimation variability for the range of flip angles (3-20°) available within the SAR limits at this short TR. In order to estimate variability and bias, the estimation algorithm of T2 and α was repeated over noised inputs. (See Methods in main text for more details). Variability was defined as the standard deviation of these estimations (for each set of true T_2 and α). Bias was defined as (the absolute of) the average difference between the estimated T_2 and the true value.

Figures S2 shows a) the variability and b) the bias of T_2 for two cases: i) using (φ_{inc1}=2°, α_{scan1}) and (φ_{inc2}=2°, $\alpha_{scan2}=2\alpha_{scan1}$), and ii) using ($\varphi_{inc1}$=3°, α_{scan1}) and (φ_{inc2}=1.5°, $\alpha_{scan2}=2\alpha_{scan1}$). As can be seen, using the latter provides lower variability – i.e. lower std($T_{2est.}$) – for a larger range of flip angles, extending the range at both the low end and the high end. The variability and bias can be examined in two presentations, as a function of (θ_1,θ_2) (the measured signal phase), and as a function of (T2,α). Both provide important insights. The (θ_1,θ_2) presentation shows ill-posed region mainly found on the left edge of the "balloon". The (T_2,α) presentation is useful for the optimal choice of parameters with respect to the T_2 and α expected in the scan.

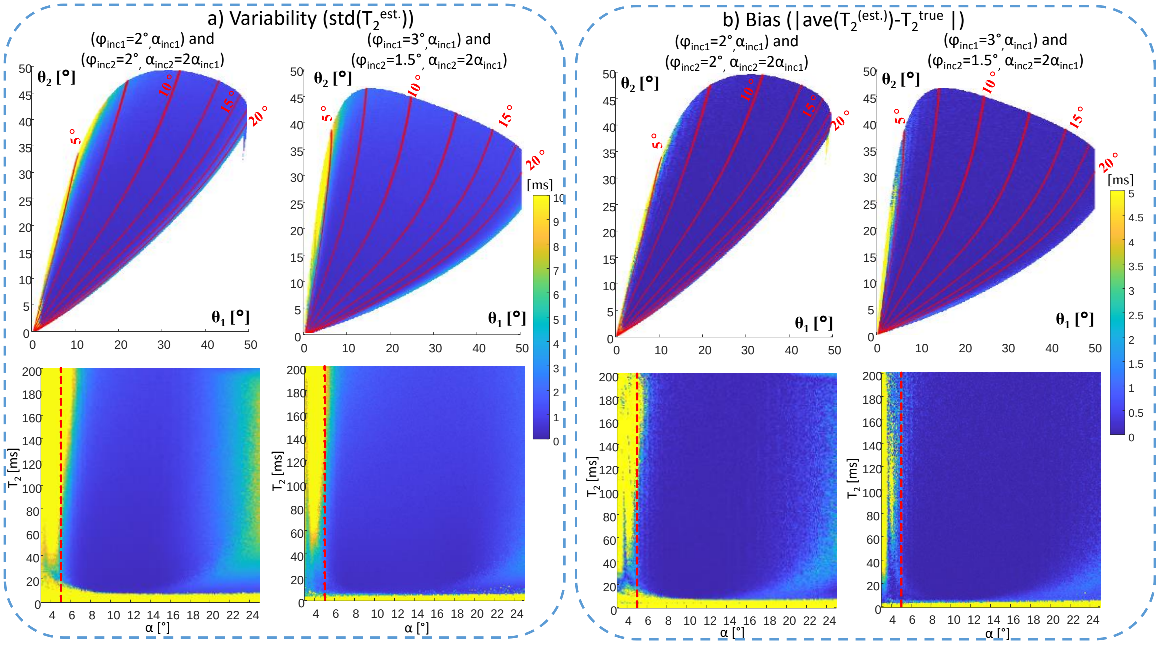

Fig. S2. Variability (a) and bias (b) for two example cases, i) (φ_{inc1}=2°, α_{scan1}) and (φ_{inc2}=2°, $\alpha_{scan2}=2\alpha_{scan1}$), and ii) ($\varphi_{inc1}$=3°, α_{scan1}) and (φ_{inc2}=1.5°, $\alpha_{scan2}=2\alpha_{scan1}$). Top, showing variability and bias as a function of (θ_1,θ_2), and bottom, the same as a function of (T_2,α). Red lines at top are equi-α lines. The red dashed lines at the bottom, at α=5°, highlight the variability and bias change at α<5°.

Three criteria were examined to help decide on the (φ_{inc1}, φ_{inc2}) combination to use with R_{FA}=2. These are shown in Figure S3: a) The average variability for 30<T_2<50 ms and 5°< α <17°; b) the minimal flip angle $\alpha_{min.}$ providing std(T2 est.)<5ms; and c) maximal flip angle $\alpha_{max.}$ providing the same. A combined minimization of the three criteria was performed and is shown in Fig.S3d. For this minimization the result of each criterion (average variability, $\alpha_{min.}$, and $\alpha_{max.}$) was linearly scaled between 0 and 1, with 0 for the lowest value and 1 for the highest in the first two criteria (average variability and $\alpha_{min.}$), while using 0 for the *highest* value and 1 for the *lowest* for the latter ($\alpha_{max.}$). The combined score of all three, which was used in the minimization, was the sum-of-squares of the three. Our final choice was a pair of scans using (φ_{inc1}=3°, α_{scan1}) and (φ_{inc2}=1.5°, α_{scan2}=2α_{scan1}) which had the lowest score in the combined minimization. It provides a good average variability for a wide range of flip angles (3.7-35°).

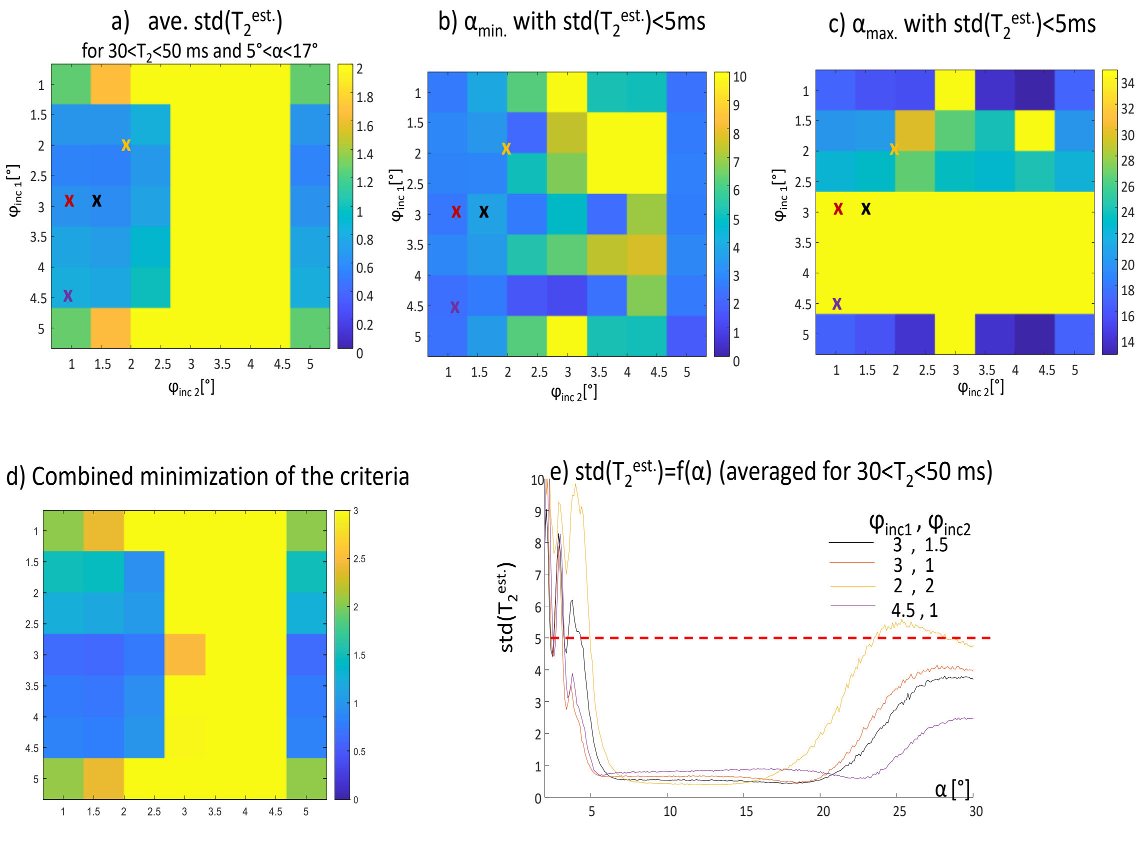

Fig. S3. Estimation variability measures for different (φ_{inc1}, φ_{inc2}) combinations with φ_{inc} in the range of 1-5°. a) Average estimation variability for 30<T_2<50 ms and 5°<α<17°; b) minimal flip angle $\alpha_{min.}$ providing std($T_2^{est.}$)<5ms; and c) maximal flip angle $\alpha_{max.}$ providing std($T_2^{est.}$)<5ms. d) Combined minimization of the three criteria in a-c) (see text) e) Shows the estimation variability as a function of α for 4 representing examples from the combinations examined (averaged over T_2 in the range of 30<T_2<50 ms).

Fig.S4 shows another three aspects considered for the scan configuration. The first being artifacts due to a strong CSF signal. Although using (φ_{inc1}=3°, α_{scan1}) and (φ_{inc2}=1°, $\alpha_{scan2}=2\alpha_{scan1}$) provides a better flip angle range — 2.4-35° according to Figs. S3b and S3c (the red x's) — in practice, the φ_{inc2}=1° scan results in a high CSF signal leading to increased residual artifacts in the proximity of the ventricles. To reduce this effect it is worthwhile to switch to φ_{inc2}=1.5° which, as shown in Fig.S4a, reduces the CSF signal. It can also be seen (Fig.S3d) that the T_2 estimation variability is generally improved — 0.52 ms for φ_{inc2}=1.5° (black line) compared to 0.63 ms for φ_{inc2}=1° (red line), for 5°<α<17°.

Fig.S4b shows that the (relative) estimation variability is insignificantly dependent on TR, in the range of interest, and so the TR can be chosen to balance SAR limitations and scan duration without further considerations. A TR of 10 ms provided a practical tradeoff.

Fig.S4c examines the effect of the R_{FA} ($\alpha_{scan2}= R_{FA}\cdot\alpha_{scan1}$) on the flip angle range. It shows that the higher the R_{FA}, the better. However, bearing in mind not to exceed 100% SAR at "Normal" level, with TR=10 ms, an R_{FA} in the range of 1.6-2 was found to provide a suitable flip angle range. In case of adopting the "First level" SAR limits, the range of the flip angles can be increased by further increasing R_{FA}.

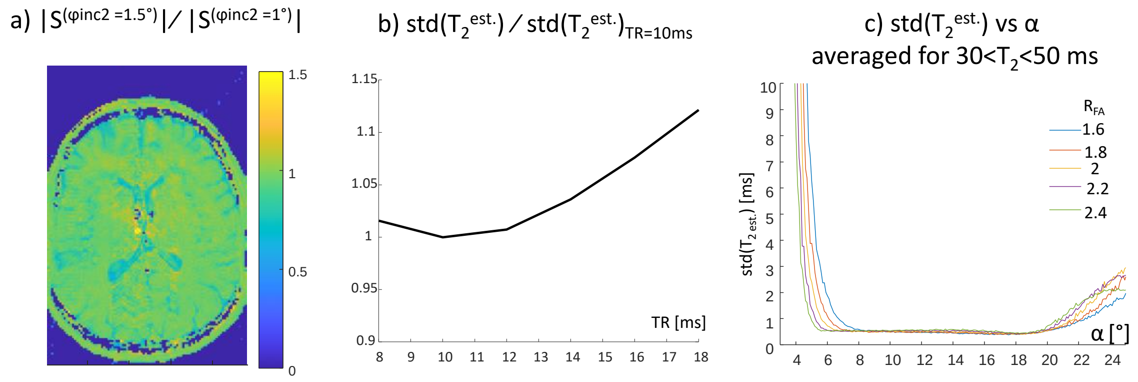

Fig. S4. Aspects to consider for the scan configuration – in terms of image quality and estimation variability. a) Choice of φ_{inc} that reduces the signal in the CSF and ventricles — φ_{inc2}=1.5° reduces the signal by ~0.7 compared to φ_{inc2}=1°. b) Relative variability as function of TR compared to TR=10ms, c) estimation variability as function of R_{FA} (estimated here with step #1).

<u>Estimation algorithm effects on variability and bias</u>

The estimation algorithm (described in the Methods section) includes an additional step to reduce the variability and bias in the range of low flip angles (<5°). Figure S5 shows a comparison of the T_2 estimation before this step and after.

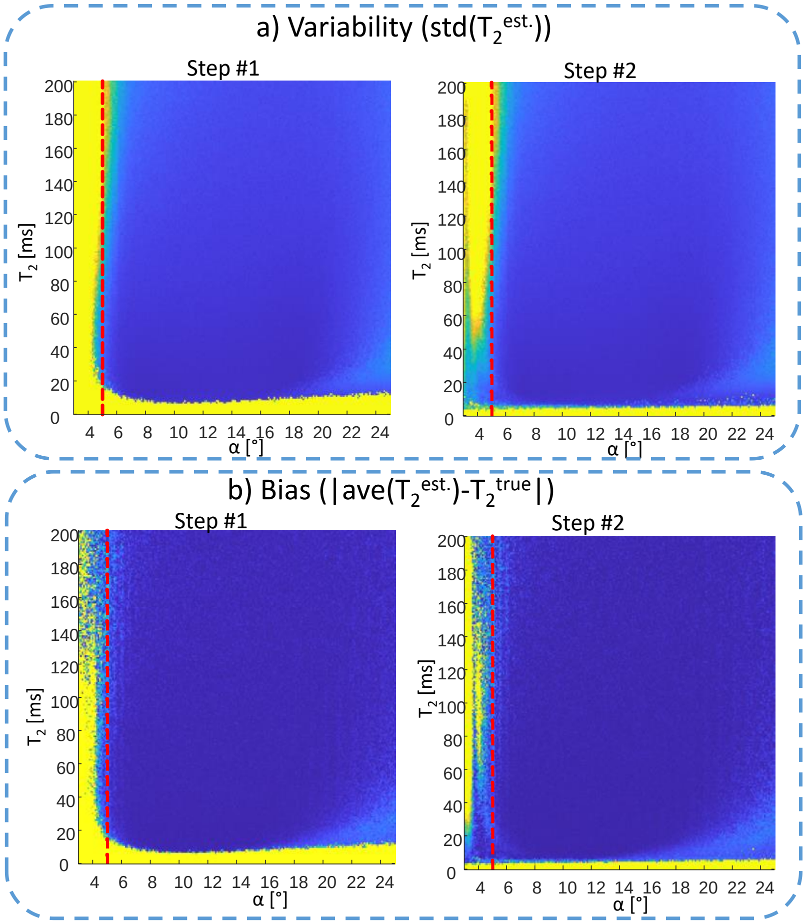

Fig. S5. Reducing the estimation variability and bias with the second iteration. (a) Estimation variability and (b) estimation bias, without (left) and with (right) the second iteration.

Finally, Fig.S6 shows the error in T_2 estimation as a function of T_1, when using two separate "dictionaries" – i.e., maps between (θ_1,θ_2) and (T_2,α) – simulated for T_1=1 sec and for T_1=2 sec. The dictionaries were simulated for (φ_{inc1}=3°, α_{scan1}) and (φ_{inc2}=1.5°, α_{scan2}=2α_{scan1}). The T_1 range examined was 0.8 to 2.5 sec.

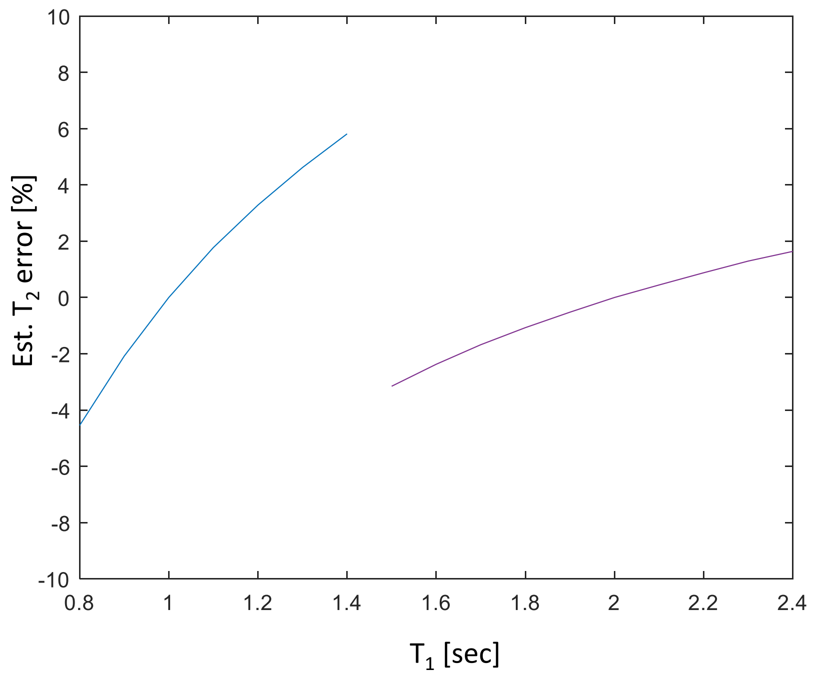

Fig. S6. Error in T_2 estimation as a function of T_1. The T_1=1s dictionary was used for 0.8<T1<1.5s and the T1=2s one used for 1.5≤T1<2.5s. The simulation was averaged for T2 in the range of 30-50 ms and α in the range of 8-20°.

S2. The effect of the gradient spoiler moment on T_2 estimation

The net gradient spoiler moment in all experiments shown was set to 0.015 [mT/m·sec] along each axis (x, y, and z), regardless of resolution. As the gradient moment affects the measured phase and so the resulting T_2 estimation, the spoiler moment was calibrated using phantom experiments to match the SE-SE T_2 estimate, which was used as a gold standard. Fig.S7a shows the effect of the spoiling moment on the T_2 estimate in five agar tubes phantoms with different T_2 values. We also examined the effect of scan resolution on the T_2 estimation with a 3D head-shaped phantom. Fig. S7b shows that there is a negligible effect — <1% on the T_2 estimation for ≥0.9 mm and <3% for ≥ 0.7 mm.

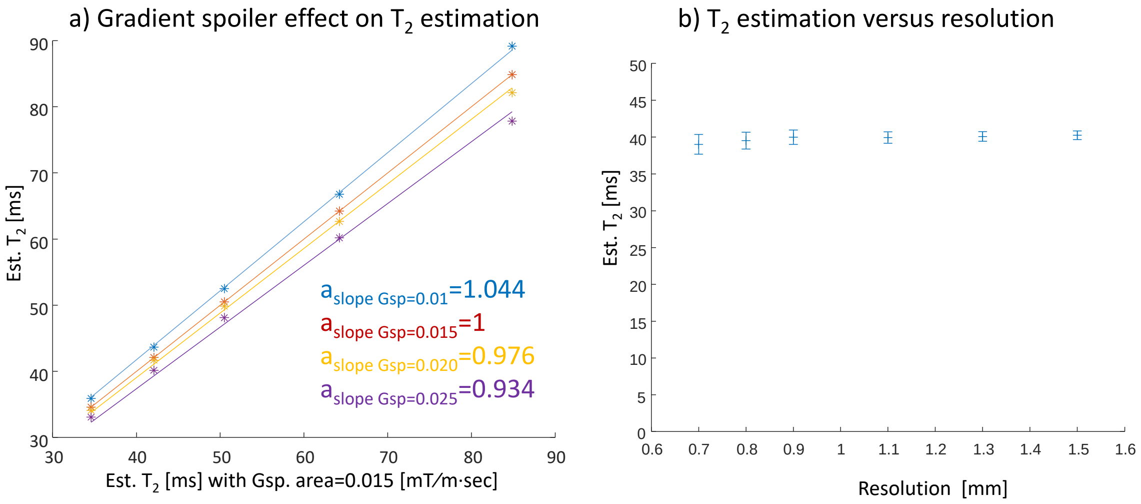

Fig. S7. Effect of the gradient spoiler moment (a) and of resolution (b) on the estimated T_2. (a) Estimated T_2 for five agar tubes (with different T_2) using different net spoiling moments. (b) The estimated T_2 in a 3D head-shaped phantom (averaged in a region shown in Fig.3) as a function of scan resolution; using a net gradient spoiler moment of 0.015 mT/m·sec.

S3. Phantom experiments

A 3D head-shaped phantom with a brain mimicking compartment was used to examine how the phase-based method copes with an RF field distribution similar to that in the brain. The uniform T_2 in the brain compartment simplified the analysis. Figure S8 shows that using only scattered data interpolation (Matlab's `scatterinterpolate()`), the estimated T_2 is much higher at places than at the central region of the phantom,

while by applying the second step, the T_2 estimation is corrected, as evident by the much more uniform T_2 distribution in the "brain" compartment of the phantom.

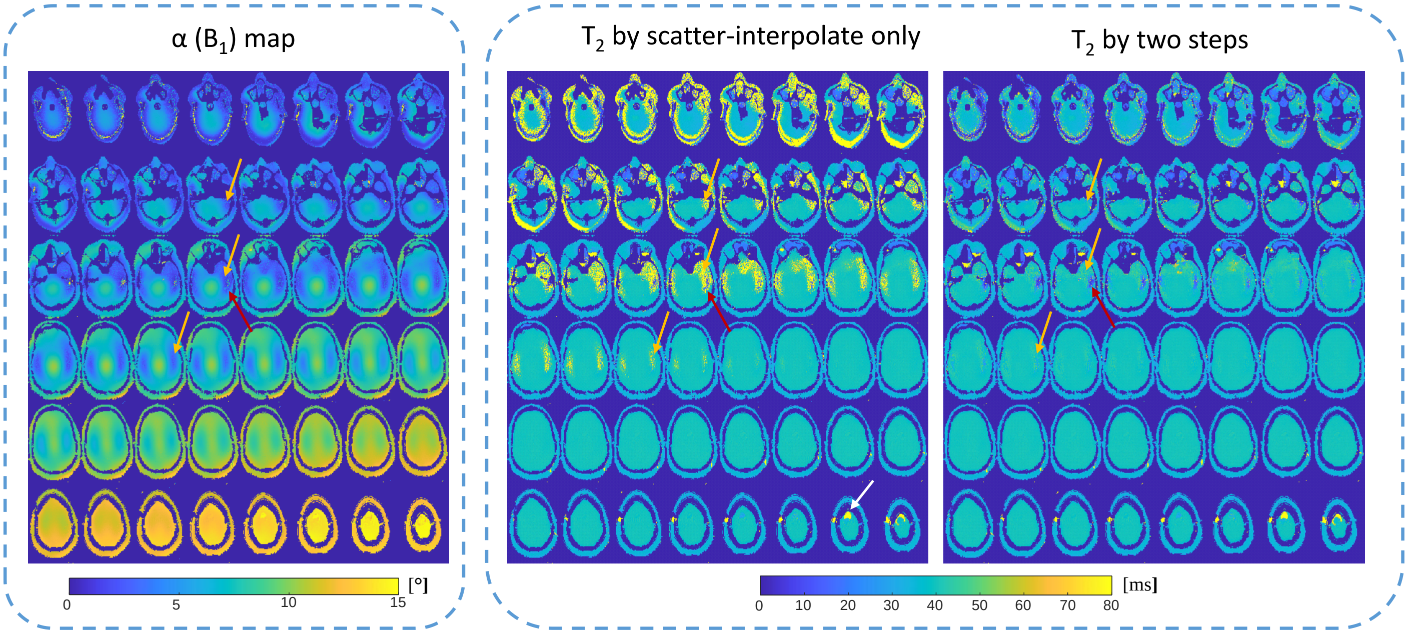

Fig.S8. α and T_2 maps (transversal slices) estimated for the 3D head-shaped phantom. From left to right, α map, T2 map using scattered data interpolation only, and the T2 map estimate including the low flip angles estimation step (see Methods in the main text). Orange arrows point to example regions with low flip angles that were recovered after the second estimation step. Red arrows point to a too-low SNR region in which case the estimation was not successful. A small region within the phantom has high T_2 values, due to trapped water in that region (see white arrow).

We also examined the T_2 estimation in case of a slab-selective acquisition. The current sequence implementation used a non-selective hard pulse for the 3D acquisition. However, for localized scans, slab-selective scans are required. Here we tested three slab-selections; starting with full brain coverage (96 partitions), then with half and a quarter of the partitions (number of partitions is the number of points acquired in the slice direction). Fig.S9 shows the estimated T_2 in two main planes. The average T_2 in the Transversal plane (in which the slice direction lies) shows negligible deviation between these cases, as well as agreeing with the non-selective case (shown in the main text, Fig. 3). The standard deviation increases with the reduction in number of partitions, as expected, due to lower SNR in this case.

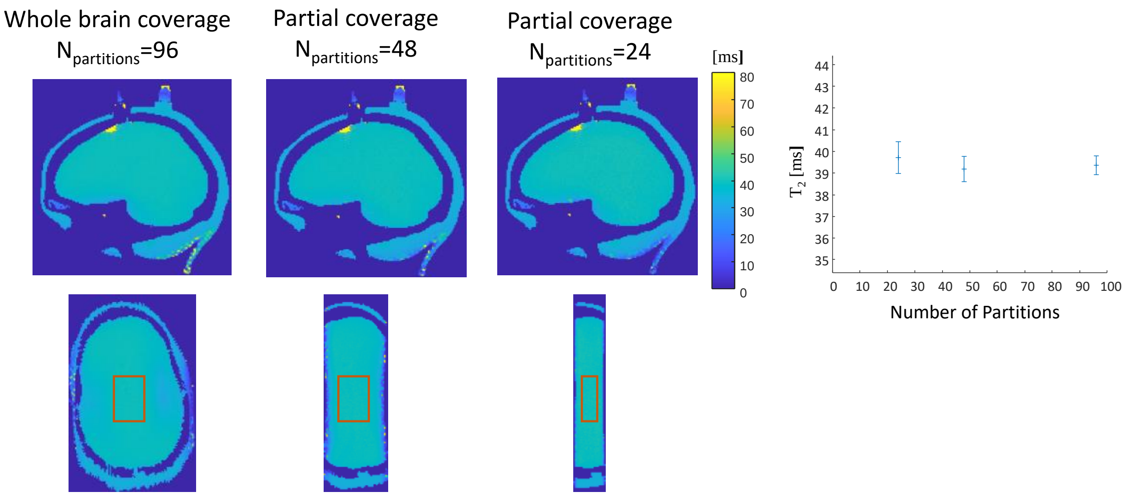

Fig.S9. Comparison of the estimated T_2 for slab-selective acquisitions – two main cross sections are shown. Different "brain" coverages with $N_{partitions}$=96 (whole-brain), $N_{partitions}$=48, and $N_{partitions}$=24 are shown on the left. The resulting estimates of T_2, average and standard deviation, are shown on the right, for voxels in the regions of interest marked by the red overlays.

S4. Human imaging

Effect of T_2 and RF distribution on raw images (before estimation algorithm)

Figure S10 shows a sample transversal slice from an in vivo "raw" image dataset, i.e., the images going into the estimation algorithm. The images are from a 0.85 mm isotropic resolution scan using (φ_{inc1}=3°, α_{scan1}) and (φ_{inc2}=1.5°, α_{scan2}=2α_{scan1}), see Methods. While the magnitude images |S1| and |S2| hardly display any T_2 weighting, the θ_1 and θ_2 images (after global phase removal) do display a strong T_2 weighting. However, the θ_1 and θ_2 images also include RF field inhomogeneity effects, especially pronounced in the θ_1 image. By using both the θ_1 and θ_2 sets in the estimation, the RF field inhomogeneity is removed and both T_2 and α maps are generated (Fig.S9b).

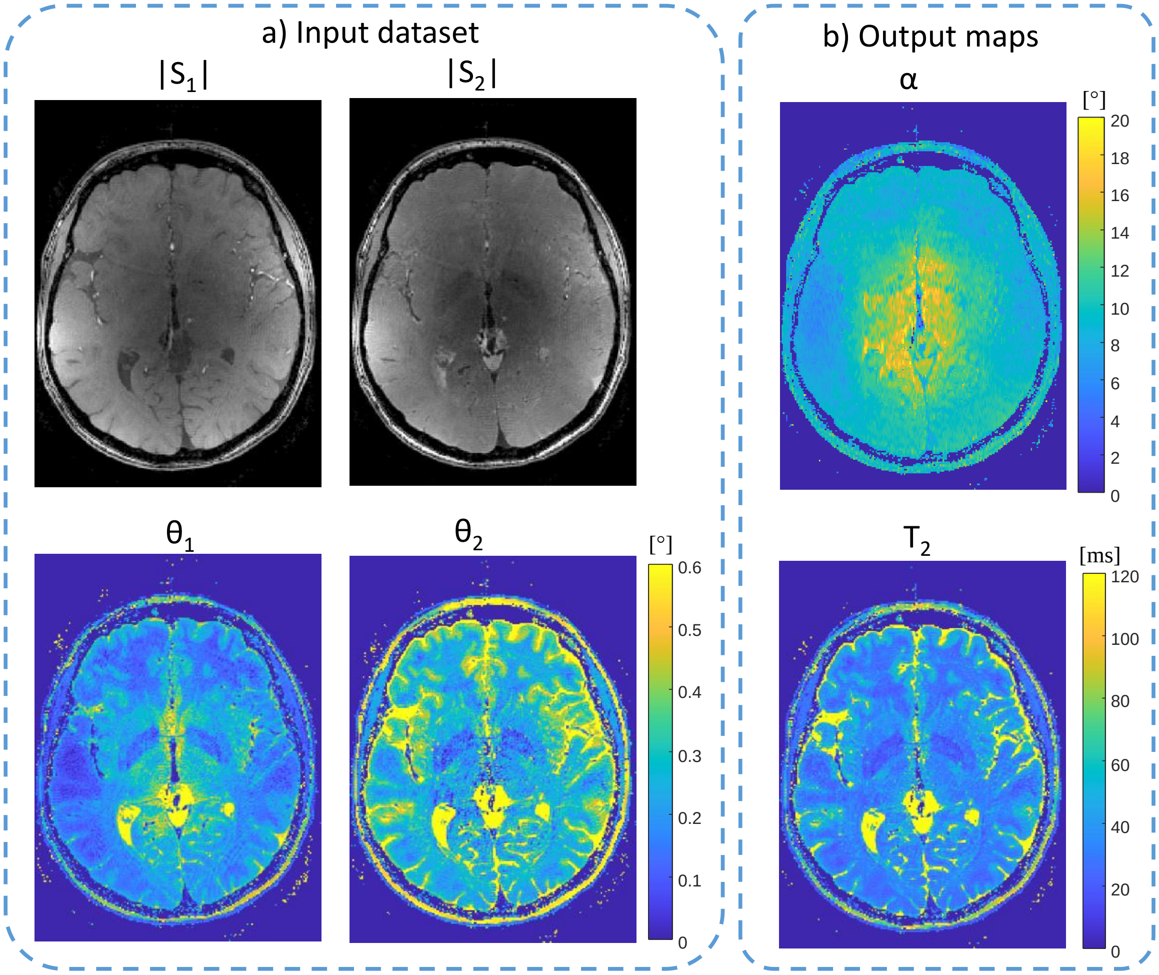

Fig. S10. An example of input dataset (a) and output maps (b), for a transversal slice. a) The magnitudes (|S1|, |S2|) and the phases (θ_1,θ_2) of the input dataset. b) The resulting α and T2 maps. The example shows a sample slice from the 0.85 mm resolution scan.

<u>The phase-based method vs. SE-SE</u>

An analysis to compare the T_2 estimation of the phase-based method and of SE-SE, the gold-standard, was performed separately for GM and WM. Many GM areas of the brain have high partial volume with either CSF and/or WM, so to reduce partial volume effects, we chose a relatively central region containing a large number of GM voxels — See Fig.S11a for a representative example in one of the volunteers. Similarly, a central region in the brain was chosen for WM voxels, as shown in Fig.S11b. Seven slices for each region were used — coronal slices for GM, and transversal slices for WM. The results are summarized in Figs.S11c as well as in Table S1.

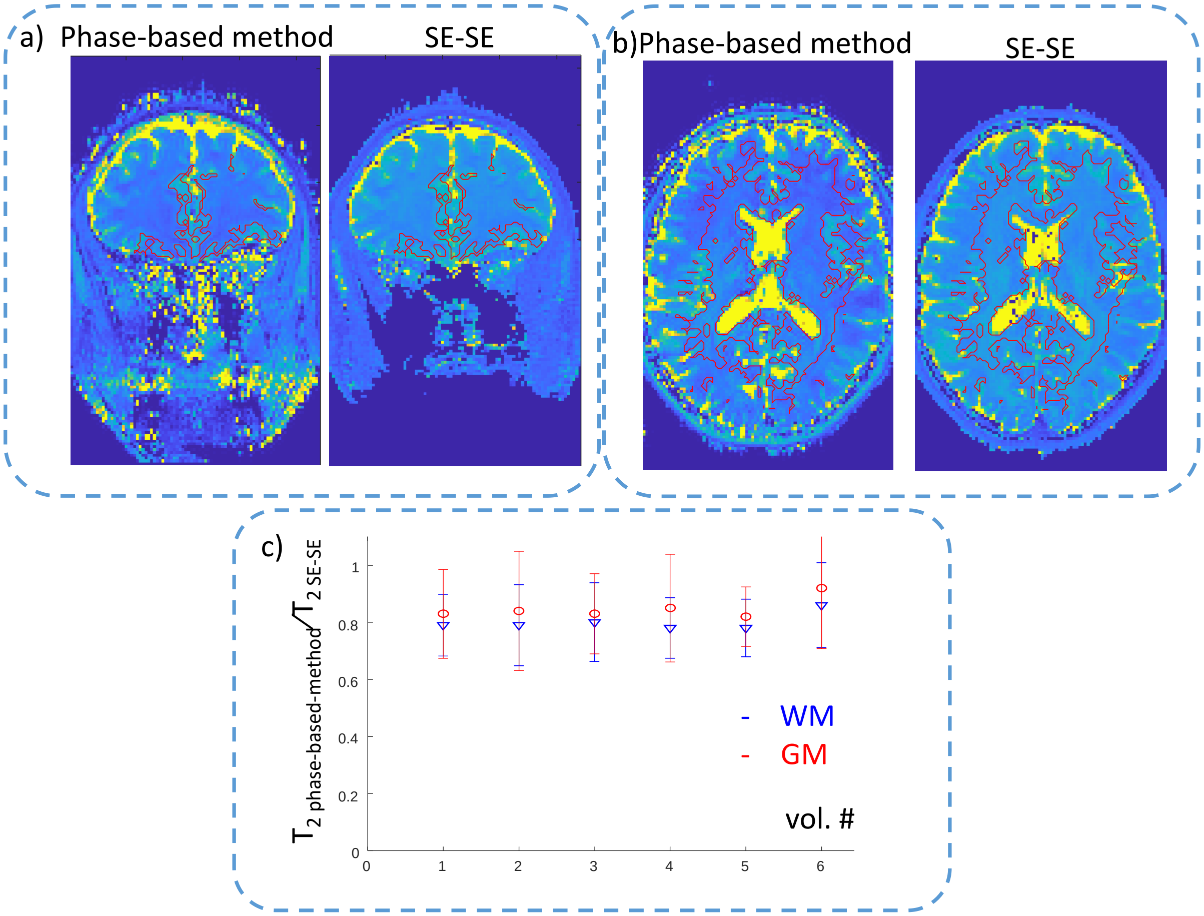

Fig. S11. Comparison of T_2 estimation between the phase-based method and SE-SE. a) A central coronal slice used to mark GM voxels, with selected voxels circumscribed by a red contour. b) A central transversal slice used to mark WM voxels, with selected voxels circumscribed by a red contour. The ratio $T_{2\ \text{phase-based-method}}/T_{2\ \text{SE-SE}}$ for WM and GM, per volunteer, shown with error bars depicting the relative deviation error (see eq.1) in c). See Table S1, as well.

Table S1. WM and GM statistics per volunteer:

Volunteer	white matter (WM)			gray matter (GM)		
	**Voxels number**	$T_{2phase\text{-}based\text{-}method}$/ $T_{2SE\text{-}SE}$	**Dev. Err[%]**	**Voxels number**	$T_{2phase\text{-}based\text{-}method}$/ $T_{2SE\text{-}SE}$	**Dev. Err[%]**
Vol. #1	12781	0.79	13.68	5051	0.83	18.75
Vol. #2	18505	0.79	17.99	3727	0.84	24.88
Vol. #3	9958	0.8	17.23	5989	0.83	16.97
Vol. #4	11576	0.78	13.64	4147	0.85	22.24
Vol. #5	18747	0.78	12.91	4762	0.82	12.73
Vol. #6	19543	0.86	17.26	2323	0.92	23.01
average	15185	0.80	15.45	4333	0.85	19.76

Denoising

Figure S12 shows sample slices of the resulting T_2 maps from human imaging acquired with 1 mm isotropic resolution (see Methods for more details). The maps on the right were found by applying denoising to the θ_1 and θ_2 phase images, before running the T_2 estimation algorithm. Denoising was performed using Matlab's pre-trained denoising convolution neural network (DnCNN). The whole brain high-resolution scans included a x5.11 acceleration, consisting of elliptical sampling and a x2 acceleration in both

phase encoding directions. Each scan was 1:13 minutes with total scan time of 4:52 minutes, for the four scans used.

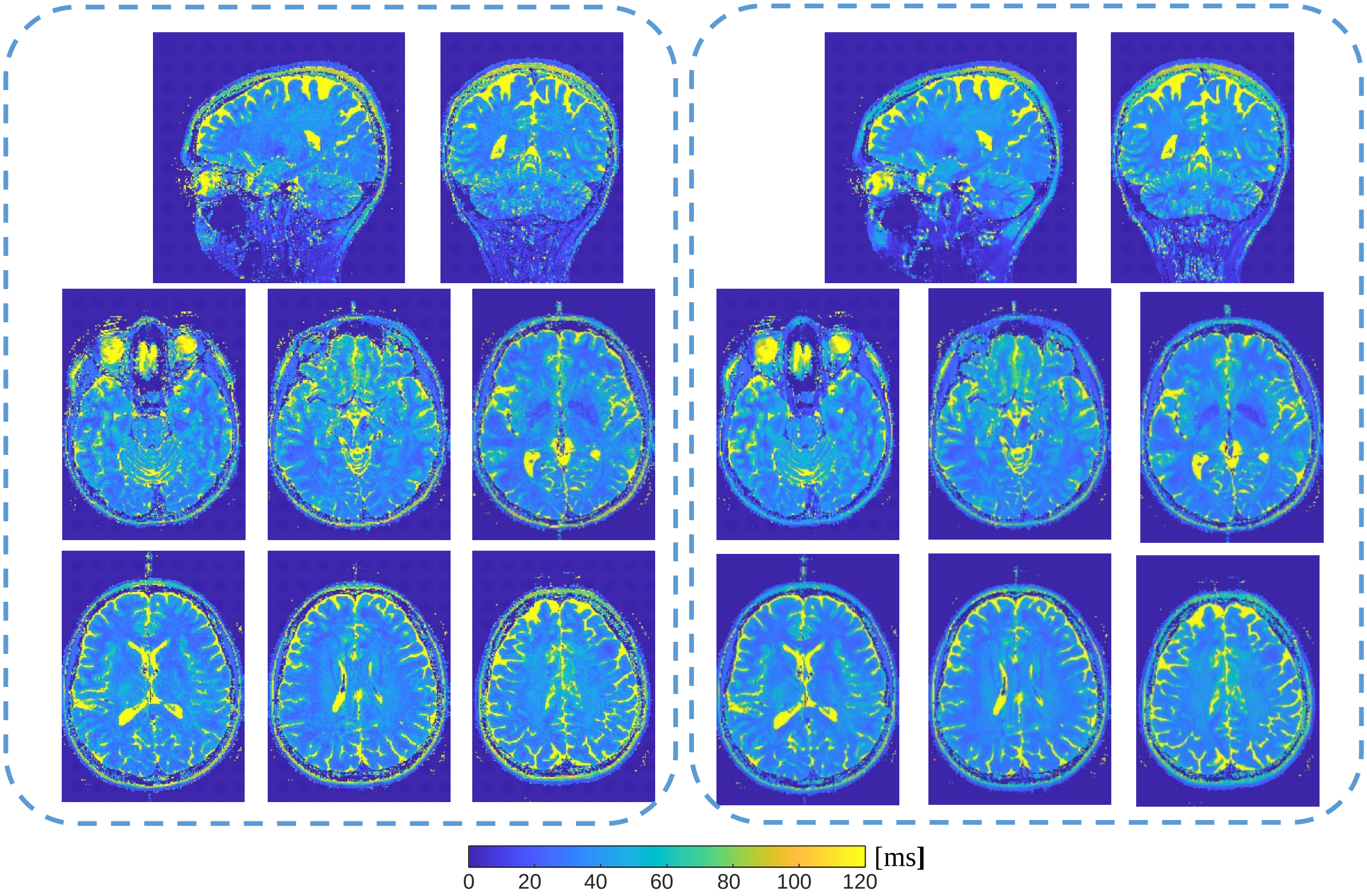

Fig. S12. **Human whole brain T_2 maps with 1 mm isotropic voxel.** Left, without denoising, and right, with denoising. Denoising was performed with a deep neural network based on a Denoising CNN (DnCNN) trained to remove Gaussian noise**.** The images show Sagittal and Coronal planes at the top row and six Axial slices every 10 mm.

B_1 (α) map from the phase-based method compared to the B_1 map acquired by the vendor’s method

When comparing the B1 maps acquired using the vendor’s supplied sequence to those acquired with the phase-based method, two reasons for deviations should be taken into account. The difference in resolution and a possible mis-estimation by the phase-based method in voxels showing CSF, especially in the ventricles. To account for this, we generated an additional B_1 map that was smoothed using a 3x3 voxels filter, after

the flip angles in the low magnitude regions in the CSF were replaced by interpolated values based on surrounding voxels. This reduced the effect of the CSF from the resulting B_1 map (shown in Fig.S13).

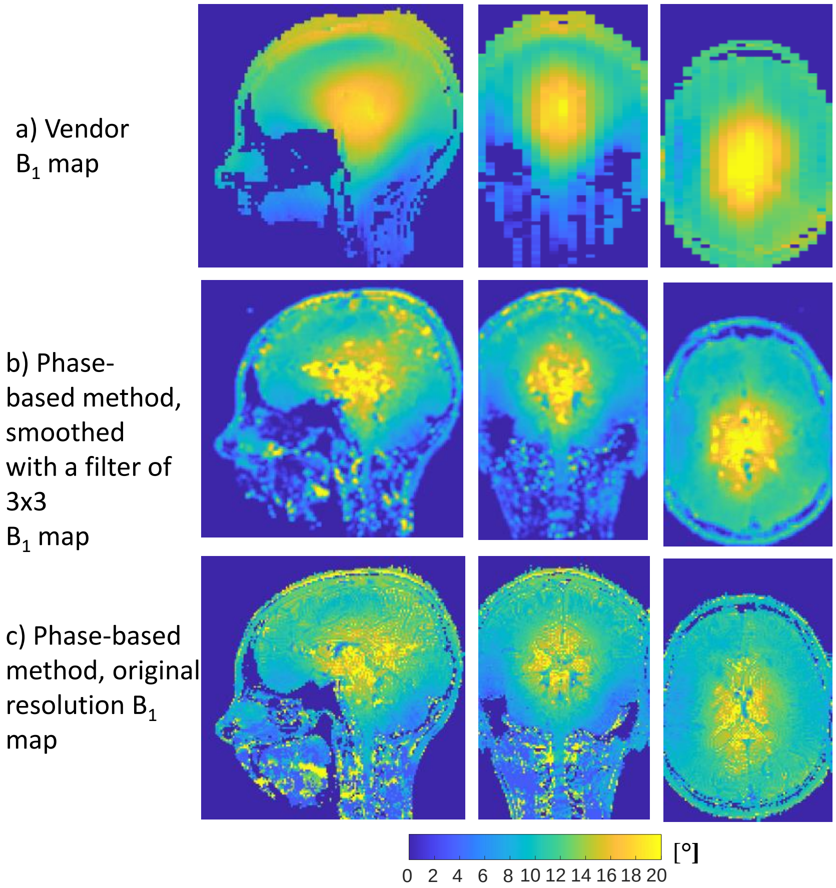

Fig. S13. Comparison between the B1 maps estimated by the vendor's scan and the phase-based method. a) The vendor's B_1 map, b) the phase-based method B_1 map after applying a 3x3 voxel filter, and c) the original, full resolution, B_1 map of phase-based method.

S5. Analysis of the phase-based method sensitivity to motion and to RF phase increment error

Simulations were performed to examine the sensitivity of the phase-based method to motion and RF phase increment errors. The T_2 range examined was 20-60 ms, and the flip angle range was 10-20°. A motion of constant velocity during the scan was simulated, similarly to Ref.[1]. Such motion results in an accumulated phase equal to $\varphi_n = n \cdot TR \cdot V \cdot 2\pi/\Delta X$, where n-is the excitation counter, V is the velocity of the motion, and ΔX is the "voxel" over which the crushers generate a 2π phase dispersion. The

simulation was performed for TR=10ms and ΔX=1.5mm. Fig. S13 shows the error in the estimated T_2 for a range of velocities, up to 0.5mm/sec.

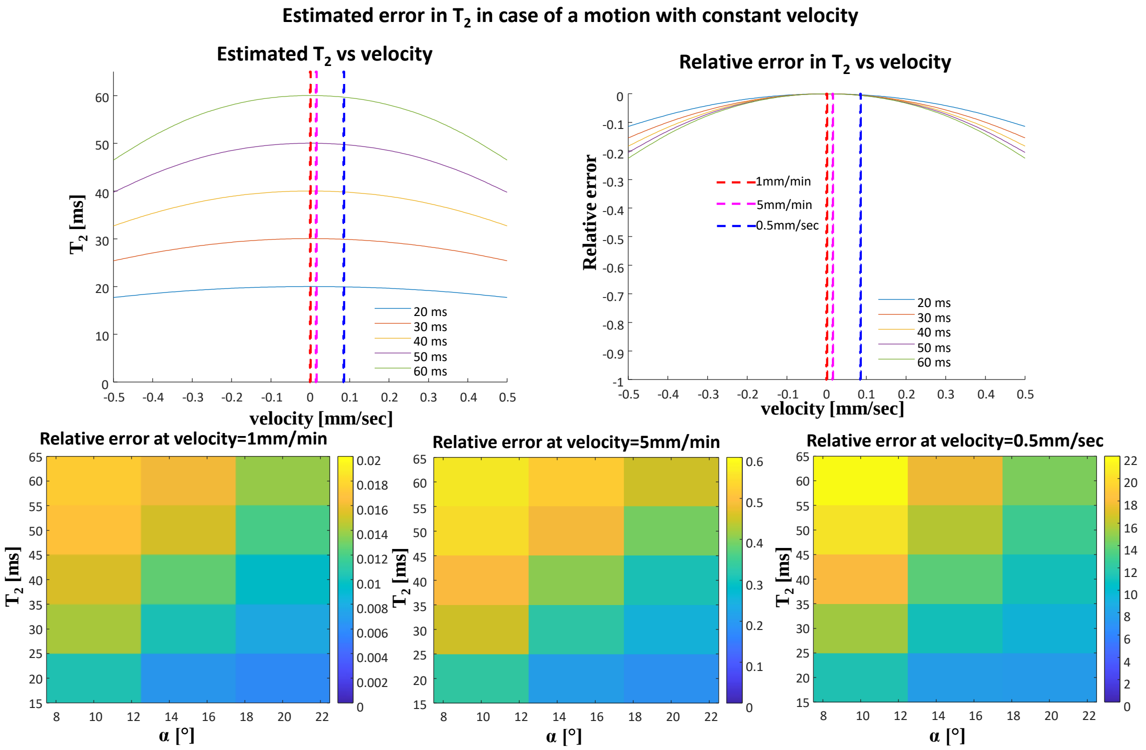

Fig. S14. Estimated T_2 error due to motion. Top – estimated T_2 and relative T_2 error for a range of velocities (up to 0.5mm/sec) and for a range of T_2 values (when α=10°). Bottom – estimated T_2 error at V=1mm/min, 5 mm/min and 0.5mm/sec (shown as vertical lines in plots above).

For a potential head movements of 1-2 voxels during the scan, i.e., up to 5mm/min (marked by the pink vertical dashed lines in Fig.S14), one can see that the error is very small, less than 1%. However, a large movement can occur in a voxel due to flow. In such a case the error of the estimated T_2 can be significant – for example, with a velocity of 0.5mm/sec the error will reach 20%.

Another potential factor for error is hardware inaccuracy of the RF phase increment or of the final actual RF phase in the RF pulse train. To examine these, simulations with RF phase increment errors of 0.1° were performed (error in φ_{inc1} only, φ_{inc2} only, and combined error). In addition, a simulation with a random normally distributed error in the actual RF phase (σ=0.2°) was added for each RF pulse. This simulation was repeated 300 times. In all simulations the T_2 range examined was 20-60 ms, and the flip angle range was 10-20°. Fig.S15 shows the results. A constant RF-phase increment error of 0.1° resulted in estimated T_2 errors <4%. The random distribution of errors

in the actual RF phase applied (σ=0.2°) resulted in a T_2 error with standard deviation

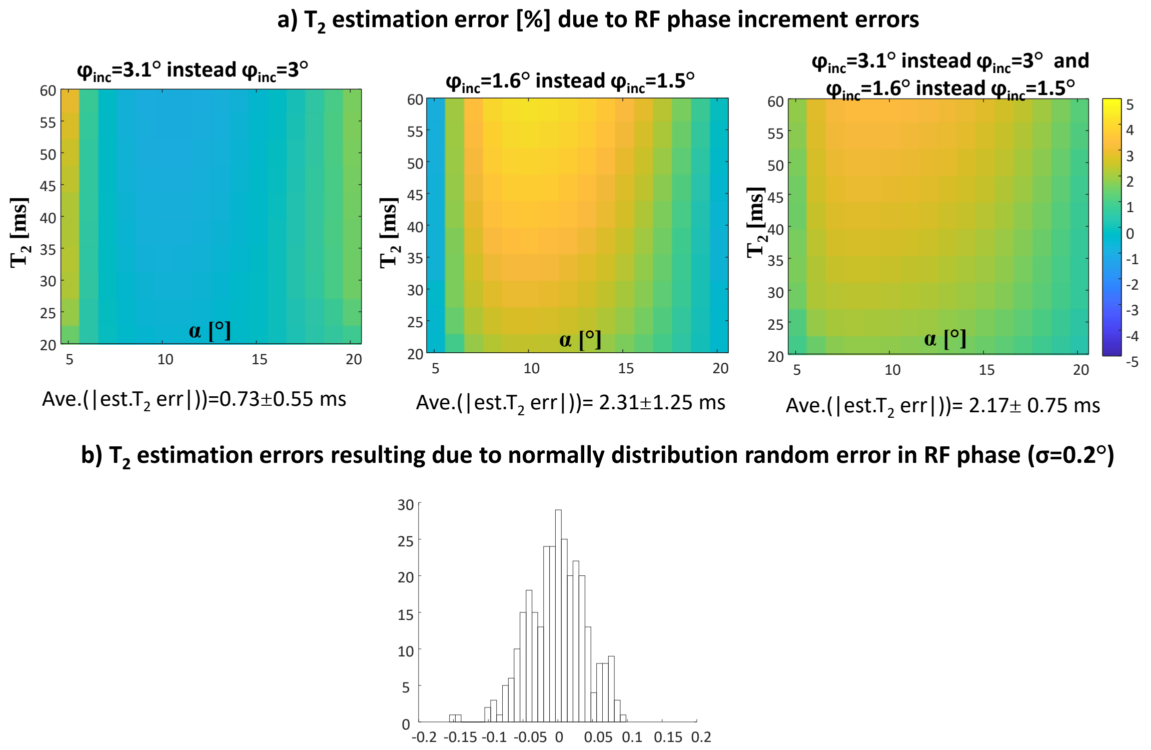

of 0.07 ms.

Fig. S15. T_2 Estimation errors due to RF phase inaccuracies. a) The estimated T_2 error due to fixed RF phase increment errors. b) A histogram of the resulting T_2 estimation errors due to normally distribution random error of the RF phases.

Movie S1 (separate file). Estimated T_2 Coronal maps of the 1 mm isotropic 3D dataset (with applied denoising).

Movie S2 (separate file). Estimated T_2 Axial maps of the 1 mm isotropic 3D dataset (with applied denoising).

Movie S3 (separate file). Estimated T_2 Sagittal maps of the 1 mm isotropic 3D dataset (with applied denoising).

Movie S4 (separate file). Estimated T_2 Coronal maps of the 0.85 mm isotropic 3D dataset (with applied denoising).

Movie S5 (separate file). Estimated T_2 Axial maps of the 0.85 mm isotropic 3D dataset (with applied denoising).

Movie S6 (separate file). Estimated T_2 Sagittal maps of the 0.85 mm isotropic 3D dataset (with applied denoising).
